# ASSET-DETR: a relation-aware multi-scale feature arbitration network for real-time weed detection in sugar beet fields

**DOI:** 10.3389/fpls.2026.1931798

**Published:** 2026-08-31

**Authors:** Haodong Liu, Linjing Wei, Pengyu Hou, Yi Hu, Yifei Ding, Yangbin Li

**Affiliations:** School of Information Science and Technology, Gansu Agricultural University, Lanzhou, China

**Keywords:** feature arbitration, few-shot domain adaptation, hypergraph computation, multi-scale feature fusion, RT-DETR, weed detection in sugar beet fields

## Abstract

**Introduction:**

At the sugar beet seedling stage, crops and weeds look alike, vary widely in scale, and appear against heterogeneous backgrounds, so a detector must judge how reliable each location and scale is, not merely extract multi-scale features.

**Methods:**

Building on RT-DETR, we propose ASSET-DETR, a relation-aware detector rebuilt at three levels: a sparse-dense attention encoder (ASSET), an attention-gated backbone (CAG-Backbone), and a hypergraph-based neck (HyperFPN). Its Hypergraph-Conditioned Multi-Feature Fusion Module (HC-MFM) conditions the fusion weights on group-level relational semantics, turning multi-scale fusion from aggregation by local statistics into feature arbitration.

**Results:**

On the public Lincoln Beet benchmark, ASSET-DETR attains 74.2% mAP50 and 51.7% mAP50-95, outperforming twelve representative detectors under a unified protocol, and improves on RT-DETR-R18 by 7.8 and 7.7 percentage points at 21.34 M parameters and 100 FPS. On external datasets, it retains about 56% of its source-domain accuracy, and on OD-SugarBeets, few-shot fine-tuning with a small amount of target-domain annotation restores performance to near source-domain levels.

**Discussion:**

ASSET-DETR is therefore a parameter-efficient candidate for object-level perception in variable-rate spraying and precision weeding, and the results underline the importance of cross-domain degradation and few-shot adaptation for this task.

## 1 Introduction

Sugar beet seedlings grow slowly and cover the ground sparsely, leaving them exposed to weed competition; uncontrolled infestations can cut yield by 40%–70%. Uniform whole-field herbicide spraying keeps this damage in check, but at the cost of chemical waste, environmental load, and mounting herbicide resistance—drawbacks that have driven the shift toward site-specific weed management (Gerhards et al., 2022). Site-specific treatment presupposes that crops and weeds can first be recognized and localized reliably. Machine-vision object detection supplies that perceptual foundation for variable-rate spraying, intelligent weeding, and autonomous field operations.

Early work on in-field weed detection relied on hand-crafted features—color, texture, and shape—paired with shallow classifiers; Kamath et al. (2020), for instance, discriminated crops from weeds using Laws’ texture masks. These pipelines are simple and interpretable, but their representational capacity is limited, and they degrade under varying illumination, cluttered soil backgrounds, shifting growth stages, and diverse weed morphologies. Convolutional neural networks (CNNs) have since become the dominant paradigm. Faster R-CNN (Ren et al., 2017) delivers accurate localization through a region proposal network, whereas one-stage detectors trade some accuracy for speed: YOLO (Redmon et al., 2016) casts detection as a single regression problem, SSD (Liu et al., 2016) predicts from multi-scale feature maps, and Focal Loss (Lin et al., 2017b) counteracts the imbalance between positive and negative samples. Hu et al. (2024) review the application of these detectors to large-scale grain production. In sugar beet fields specifically, WeedNet-R (Guo et al., 2023b) strengthens the feature representation of RetinaNet, and Gao et al. (2020) detect field bindweed with deep CNNs.

CNN detectors nonetheless inherit two structural constraints: local convolutional receptive fields and non-maximum suppression (NMS) as post-processing. Both become problematic at the sugar beet seedling stage. Seedlings closely resemble broad-leaved weeds such as goosefoot and amaranth, and plants grow densely, occlude one another, and span a wide range of scales. Local texture cues and adjacent-level feature fusion then produce false detections of look-alike objects and missed detections of small ones, while NMS erroneously suppresses tightly neighboring boxes. DETR (Carion et al., 2020) removes the NMS stage entirely by casting detection as set prediction with bipartite matching. Its slow convergence prompted two influential refinements: Deformable DETR (Zhu et al., 2021) attends to a sparse set of sampling points, and DINO (Zhang et al., 2023) adds contrastive denoising training and mixed query selection, improving both convergence speed and accuracy.

RT-DETR (Zhao et al., 2024) brought this end-to-end paradigm to the real-time regime through an efficient hybrid encoder and uncertainty-minimal query selection. Agricultural adaptations followed quickly: PHRF-RTDETR (Jin et al., 2025) targets rice fields, EMCF-RTDETR (Yang et al., 2025) adds efficient multi-scale channel enhancement, and WS-RTDT (He et al., 2025) improves crop–weed discrimination with wavelet convolution and sparse cubic attention. Real-time end-to-end detection is thus mature enough to support in-field perception. We adopt RT-DETR as our base framework and tailor it to the specific conditions of sugar beet fields.

Even so, directly applying a general-purpose real-time detection framework to sugar beet fields still faces three difficulties. First, complex field backgrounds contain interference from soil, withered leaves, shadows, and crop residues; the global Softmax attention adopted by the RT-DETR encoder tends to disperse responses to non-target regions, and its feed-forward network transforms features only along the channel dimension, lacking explicit modeling of two-dimensional spatial structures such as edge contours, which makes it difficult for the encoder to focus stably on the key regions of crops and weeds. Second, sugar beet seedlings and weeds are highly similar in color, leaf shape, and scale, so a ResNet backbone relying solely on fixed local receptive fields can hardly capture local texture, long-range dependencies, and cross-scale contextual information simultaneously. Finally, existing multi-scale feature fusion methods mostly rely on the summation, concatenation, or pairwise relationship modeling of adjacent levels; although they can enhance scale adaptability, they lack an explicit judgment of the relative reliability of features at different scales in the current scene and thus struggle to characterize cross-scale high-order semantic relationships adequately.

Each of these difficulties corresponds to an active line of work. Standard multi-head self-attention (Vaswani et al., 2017) assigns nonzero weight to every position, so its cost grows quadratically with sequence length and its responses disperse over clutter—a weakness that became conspicuous once the architecture was brought to dense visual prediction (Dosovitskiy et al., 2021). Sparsification addresses this by restricting the attention field, through shifted local windows (Liu et al., 2021), learnable token sparsity (Roh et al., 2022), a small budget of latent tokens (Gao et al., 2024), or smooth ReLU² gating (Hua et al., 2022). Purely sparse attention, however, can discard global context; Zhou et al. (2024) therefore fuse sparse and dense branches adaptively, the design our ASSA builds on. On the feed-forward side, convolutional spatial priors placed inside the transformation sharpen the encoding of edges and local geometry (Wu et al., 2021; Shi, 2024), which motivates our SEFN.

For cross-scale modeling, pyramid attention (Wang et al., 2021) and multi-scale convolutional attention (Guo et al., 2022) inform the design of our CAG-Backbone, whose MSMHSA decouples the original-resolution query from multi-scale keys and values. For fusion, FPN (Lin et al., 2017a) established the top-down paradigm, AFPN (Yang et al., 2023) narrows the semantic gap between non-adjacent levels through progressive fusion, MSE-FPN (Guo et al., 2023a) adds multi-scale semantic enhancement, and Fog-UAVNet (Dong et al., 2026) adapts the multi-scale prior to degraded imaging conditions. All of these remain grounded in pairwise relationships between levels, and none forms an explicit judgment of how reliable each scale is in the current scene. Hypergraphs supply the missing primitive: because a hyperedge connects any number of nodes, they model high-order group relations natively (Feng et al., 2019; Gao et al., 2023), and vision applications now span abnormal-cell detection (Li et al., 2026b), real-time object detection (Feng et al., 2025), and cross-modal UAV detection (Li et al., 2026a). In all of these the hypergraph acts as a feature-enhancement or interaction component, and its group-level semantics have not been used to condition multi-scale fusion weights. That coupling is the entry point of this work.

To address these issues, we propose ASSET-DETR, a relation-aware multi-scale feature arbitration network for real-time weed detection in sugar beet fields. Our premise is that this task is not solved by extracting more features. What a detector needs, under cluttered backgrounds, look-alike objects, and wide scale variation, is a way to judge which spatial locations, scale levels, and cross-scale relationships to trust. We call this judgment feature arbitration: instead of aggregating multi-scale features with weights derived from each branch’s own local statistics, the network weighs the competing branches against shared relational evidence and modulates their contributions by inferred reliability. Guided by this idea, we redesign RT-DETR at three levels—the encoder, the backbone, and feature fusion.

At the encoder level, we propose the Adaptive Sparse-dense Spatially-Enhanced Transformer encoder (ASSET). It draws on the dual-branch attention of Zhou et al. (2024) and on convolutional gated feed-forward designs (Wu et al., 2021; Shi, 2024). A sparse–dense dual-branch attention suppresses the dispersion of attention over cluttered backgrounds, and a spatially enhanced feed-forward network injects two-dimensional structural priors into the feature transformation, sharpening the encoding of edges, contours, and local geometry.

At the backbone level, we propose the CSP Attention-Gated Backbone (CAG-Backbone)—named for the CSP framework, the multi-scale attention, and the gated linear unit that constitute it—to lift the single-scale limitation of ResNet. Building on multi-scale attention designs such as PVT (Wang et al., 2021) and SegNeXt (Guo et al., 2022), it embeds multi-scale multi-head self-attention and a convolutional gated linear unit within the CSP framework, so that local texture, long-range dependencies, and channel selection are modeled jointly—precisely the combination needed to separate sugar beet seedlings from morphologically similar weeds.

At the fusion level, we propose HyperFPN and, within it, the Hypergraph-Conditioned Multi-Feature Fusion Module (HC-MFM). Whereas Hyper-YOLO (Feng et al., 2025) and HyperDet (Li et al., 2026a) use the hypergraph purely as a feature-enhancement component, HC-MFM feeds the cross-scale group-level semantics recovered by hypergraph modeling into the generation of the fusion weights themselves. Multi-scale fusion thereby shifts from FPN-style aggregation (Lin et al., 2017a) to reliability arbitration under high-order relational constraints.

Finally, we systematically validate ASSET-DETR on the LB source domain, under a zero-shot evaluation on external sugar beet field datasets, and under a few-shot target-domain adaptation protocol, and we assess its effectiveness and applicability boundaries from the perspectives of comparison with mainstream detectors, module ablation, visualization analysis, cross-domain degradation, and failure cases. The experimental results provide supporting evidence for the proposed relation-aware feature arbitration mechanism, and further reveal the importance of cross-domain generalization and few-shot adaptation in weed detection in sugar beet fields.

## 2 Materials and methods

### 2.1 Datasets

#### 2.1.1 Source dataset and basic characteristics

We adopt the public sugar beet field weed detection benchmark—the Lincoln Beet dataset (LB)—as the experimental carrier. LB was released by Salazar-Gomez et al. (2021) and has been widely used as an evaluation benchmark owing to its diverse field scenes and rigorous annotations.

LB was collected by the L-CAS team at the University of Lincoln, UK, in three typical sugar beet fields; after frame sampling and redundancy removal, it yields 4,402 RGB images of 1,920×1,080, covering the cotyledon stage to the multiple-true-leaf stage, various soil backgrounds, and illumination at different times of day, and is therefore highly representative of real conditions (with an object density of approximately 8.9 instances per image). As shown in **Supplementary Figure 1**, the objects include sugar beet plants and common accompanying weeds such as barnyard grass, foxtail, goosefoot, and reed; these weeds are visually similar to sugar beet in leaf shape, leaf color, and spatial distribution, which allows the model’s ability to discriminate fine-grained crop–weed differences to be tested.

#### 2.1.2 Class definition and annotation specification

For a variable-rate spraying decision, the most fundamental information is whether an object is a weed rather than its specific species. We therefore follow the original LB annotation specification and adopt a two-class scheme of sugar beet crop (crop) and field weed (weed), merging all non-sugar-beet plants into the weed class; this both fits the requirements of precision weeding and avoids the sample imbalance of fine-grained multi-class settings.

The annotations adopt the normalized quintuple $\left(c,x_{c},y_{c},w,h\right)$ in YOLO format. The original publisher corrected missed labels, mislabels, and bounding-box tightness issues through multiple rounds of manual verification and cross-validation. The dataset contains 4,402 images and 39,246 instances (16,399 crop and 22,847 weed, a ratio of approximately 1:1.39, a mild imbalance); Focal Loss is used during training to mitigate the imbalance, and the statistics are given in **Table 1**.

**Table 1:** Statistics of the dataset used in the experiments.

Dataset	Source country	No. of images	Resolution (px)	Total instances (crop/weed)
Lincoln Beet (LB)	UK	4,402	1,920 × 1,080	39,246 (16,399/22,847)

#### 2.1.3 Data split and data augmentation

For the data split, we divide the dataset into training, validation, and test sets at a ratio of 7:2:1. The random seed is fixed to 42 during splitting to ensure reproducibility of the split. For data augmentation, multiple strategies are combined online during training, each targeting a different difficulty of the sugar beet field scene: Mosaic stitches four images together with probability 1.0, enriching the per-image object density and context and improving the robustness to small objects and densely growing scenes; MixUp linearly blends images with probability 0.10 to alleviate overfitting and smooth the class decision boundary; HSV color jittering simulates different illumination and soil color differences in the field; random horizontal flipping with probability 0.5 enhances orientation invariance; and random affine transformation simulates variations in shooting viewpoint and object scale. Considering that Mosaic introduces synthetic artifacts in the late training stage and interferes with fine refinement, it is disabled in the last 10 epochs; the validation and test sets maintain their original distributions without any augmentation.

#### 2.1.4 External datasets and class harmonization

OD-SugarBeets (Chebrolu et al., 2017) is built upon SugarBeet2016 and contains 4,815 images (with an official train/val/test split of 3,466/385/964) with original classes crop and weed; to be consistent with the source domain, crop is mapped to sugar beet and weed is kept unchanged.

PhenoBench (Weyler et al., 2024) provides 1,024×1,024 top-view RGB images and instance masks; because the official test annotations are not publicly available, we use the official validation set as the external evaluation set and generate detection boxes from plant_instances and semantics, mapping crop/partial-crop to sugar beet and weed/partial-weed to weed. The splits, classes, and post-conversion box counts of the two external datasets are given in **Supplementary Table 5**.

Note: Because the official PhenoBench test annotations are not publicly available, external evaluation is conducted only on the official validation set.

### 2.2 Overall architecture of ASSET-DETR

Sugar beet fields confront a detector with three difficulties at once: crops and weeds that look alike, objects that span a wide range of scales, and backgrounds that vary from bare soil to straw residue. General-purpose detectors struggle to hold accuracy and efficiency together under these conditions. RT-DETR strikes a reasonable compromise through its hybrid encoder and IoU-aware query selection, but three of its components limit it here. The ResNet backbone sees only local receptive fields at a single scale. The AIFI encoder relies on global Softmax attention, which disperses over clutter, and on a point-wise feed-forward that ignores spatial structure. The CCFF neck fuses adjacent levels pairwise and cannot express higher-order dependencies. We map these three weaknesses onto three levels of the architecture—spatial attention, scale context, and fusion reliability—and address them with ASSET, CAG-Backbone, and HC-MFM, respectively.

**Figure 1:**
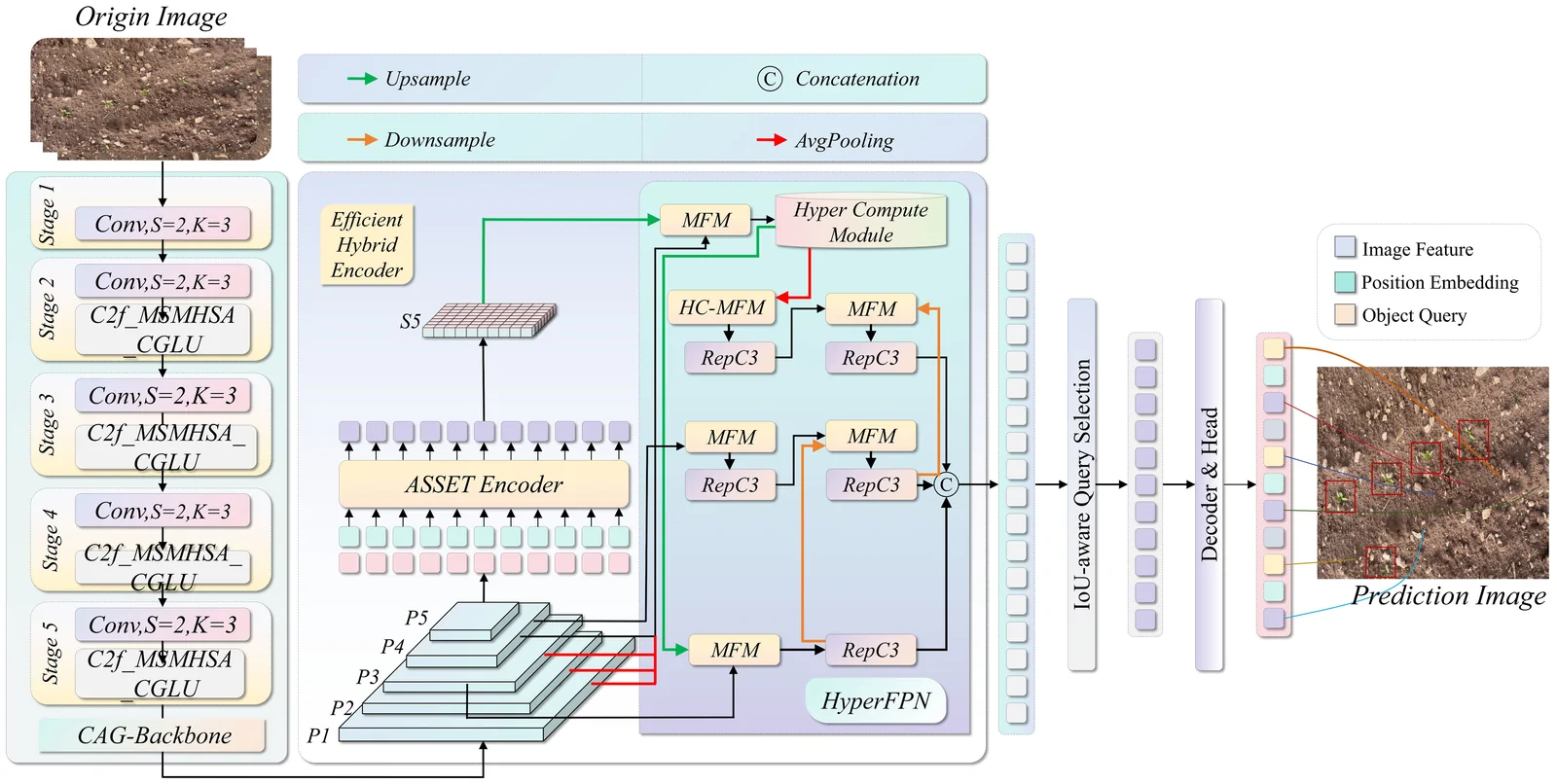
Overall network architecture of ASSET-DETR. The fusion module that receives the pooled hypergraph output is HC-MFM, whose internal structure and hypergraph-conditioning pathway are detailed in **Figure 4**; the remaining fusion modules follow the standard MFM formulation of Equations 2–15.

ASSET-DETR rebuilds the feature pathway of RT-DETR at all three levels (**Figure 1**). In the backbone, CAG-Backbone replaces the ResNet residual stages with C2f_MSMHSA_CGLU blocks, combining local–global modeling with adaptive channel refinement inside the CSP framework. In the encoder, ASSET substitutes adaptive sparse self-attention (ASSA) and a spatially enhanced feed-forward network (SEFN) for the standard AIFI components, keeping global modeling while tightening attention focus and adding spatial-geometric expressiveness. In the neck, HyperFPN captures the high-order semantic associations among multi-scale nodes with a hypergraph computation module, and HC-MFM conditions the fusion weights on the resulting group-level semantics—converting fusion from plain aggregation into relation-aware feature arbitration.

### 2.3 ASSET encoder

The AIFI module of RT-DETR is a standard Transformer encoder layer, and it carries two weaknesses into the field. First, globally Softmax-normalized self-attention over-attends to irrelevant regions—soil, withered leaves, shadows—so its responses disperse. Second, the feed-forward network transforms features channel-wise only, blind to spatial structure. The ASSET encoder layer replaces both components: ASSA restores attention focus, and SEFN adds spatial-context awareness, while global modeling is preserved.

**Figure 2:**
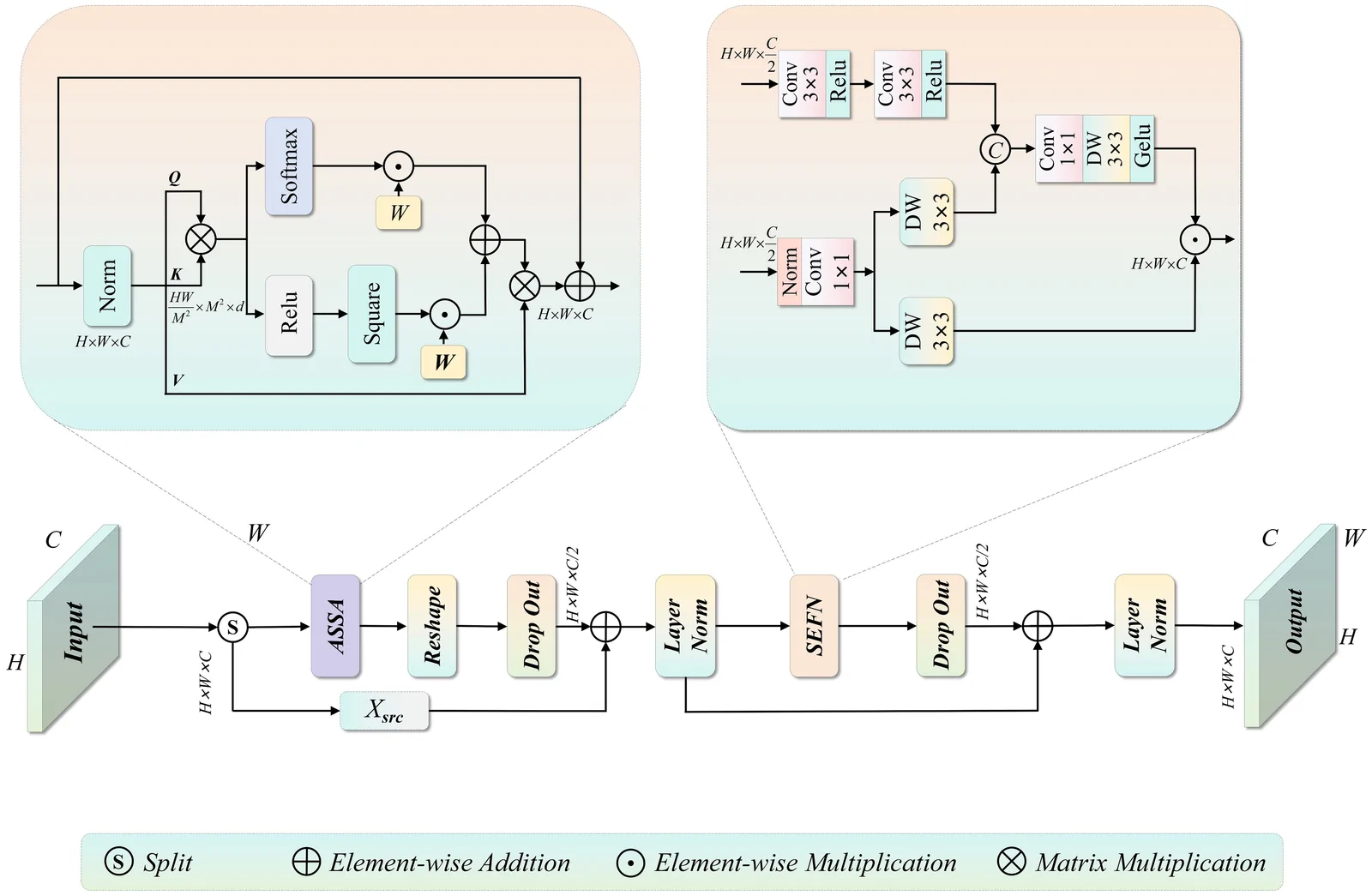
Structure of the ASSET encoder.

ASSET follows a Post-Norm residual structure, in which layer normalization is applied after each residual addition. Given an input $X\in {\mathbb{R}}^{B\times N\times C}$ (where N=H×W is the number of flattened spatial positions), a single-layer encoder can be abstracted as the following composition of operators:


ASSET≜LN∘(Id+D∘SEFN)∘LN∘(Id+D∘ASSA)

where Id denotes the identity mapping, ∘ denotes operator composition, and D and LN denote the Dropout and layer-normalization operators, respectively. Equations 1, 2 shows that ASSET is formed by cascading two Post-Norm residual operators: ASSA realizes the adaptive fusion of sparse–dense dual-branch attention, and SEFN injects spatial-structure priors into the channel transformation; together they output high-quality features that possess both spatial focus and local-texture expression. The overall structure of ASSET is shown in **Figure 2**.

Adaptive sparse self-attention (ASSA) constructs a sparse–dense dual-branch adaptive fusion to alleviate the dispersion of global Softmax attention. The input is projected by a unified linear projection into Q, K, and V, which are reshaped into multiple heads; on the scaled dot-product scores S, each head computes two branches in parallel: the dense branch preserves global context via Softmax, while the sparse branch zeros out weakly correlated scores through a rectified-square operator ${\left(\cdot \right)}_{+}^{2}$ to filter background noise. The two branches are fused with learnable scalars $w_{1}$ and $w_{2}$ and re-normalized by a row-normalization operator $N_{\text{row}}$:

(1) $$S=\frac{QK^{⊤}}{\sqrt{d_{h}}},\;Q,K,V=\text{Split}(XW_{qkv})$$

(2) $$A=N_{\text{row}}(w_{2}\;\sigma (S)+w_{1}\;{\left(S\right)}_{+}^{2})$$

where $Q,K,V\in {\mathbb{R}}^{Bh\times N\times d_{h}}$, $d_{h}=C/h$, and $W_{qkv}\in {\mathbb{R}}^{C\times 3C}$; σ(·) denotes Softmax normalization, and ${\left(\cdot \right)}_{+}^{2}$ denotes ReLU rectification followed by element-wise squaring. Both $w_{1}$ and $w_{2}$ are initialized to 1 and are adaptively adjusted during training to seek the optimal sparse–dense balance. Compared with hard-threshold strategies such as Top- k, this soft sparsification zeros out negatively correlated scores while keeping the gradient differentiable. The final attention output is O=AV, mapped back to the original dimension by a linear projection.

The spatially enhanced feed-forward network (SEFN) introduces spatial-structure information into the feed-forward transformation to strengthen local geometric expression. After the input is reshaped into a two-dimensional feature map, a spatial gating signal is generated through a spatial-aware pathway composed of average pooling, two groups of 3×3 convolutions, and bilinear upsampling; meanwhile, the input is split by linear projection into a main path U and a gating path G, where G is multiplied element-wise by the spatial gating signal to inject the spatial prior, and is then modulated by U through gating and restored in dimension by a linear projection:

(3) $$\text{SEFN}(X)=W_{\text{out}}(U⊙\text{GELU}(G⊙\Phi_{s}(X))),\;U,G=XW_{\text{in}}$$

where $U,G=XW_{\text{in}}\in {\mathbb{R}}^{B\times N\times 2C}$ is the content–gating dual-branch projection, and $W_{\text{out}}\in {\mathbb{R}}^{C\times C}$ is the output projection; ⊙ denotes element-wise multiplication; the spatial-prior operator $\Phi_{s}≜\text{Up}\circ {\text{Conv}}_{3\times 3}^{\left(2\right)}\circ P$ is composed successively of average pooling, two layers of 3×3 spatial convolution, and upsampling. This spatial gating mechanism enables the feed-forward network to simultaneously exploit local spatial-structure information during the channel-wise nonlinear transformation, remedying the deficiency of the standard FFN in ignoring spatial topology, which is particularly important for capturing weed edge contours and spatial distribution patterns.

Taken together, the dual-branch fusion of ASSA suppresses background noise and focuses on weed-relevant locations while preserving global perception, and the spatially gated feed-forward of SEFN injects geometric awareness into the channel transformation; together they enable ASSET to output high-quality features that possess both spatial focus and local expressiveness.

### 2.4 CAG-backbone

The ResNet backbone of RT-DETR sees the field through fixed local receptive fields at a single scale. Visually similar crops and weeds are therefore easily confused, and objects at unusual scales receive little representational support. CAG-Backbone addresses both problems by replacing each ResNet residual stage with a C2f_MSMHSA_CGLU block. Multi-scale multi-head self-attention and a convolutional gated linear unit, embedded in the CSP framework, give the backbone joint local–global modeling and adaptive feature refinement.

**Figure 3:**
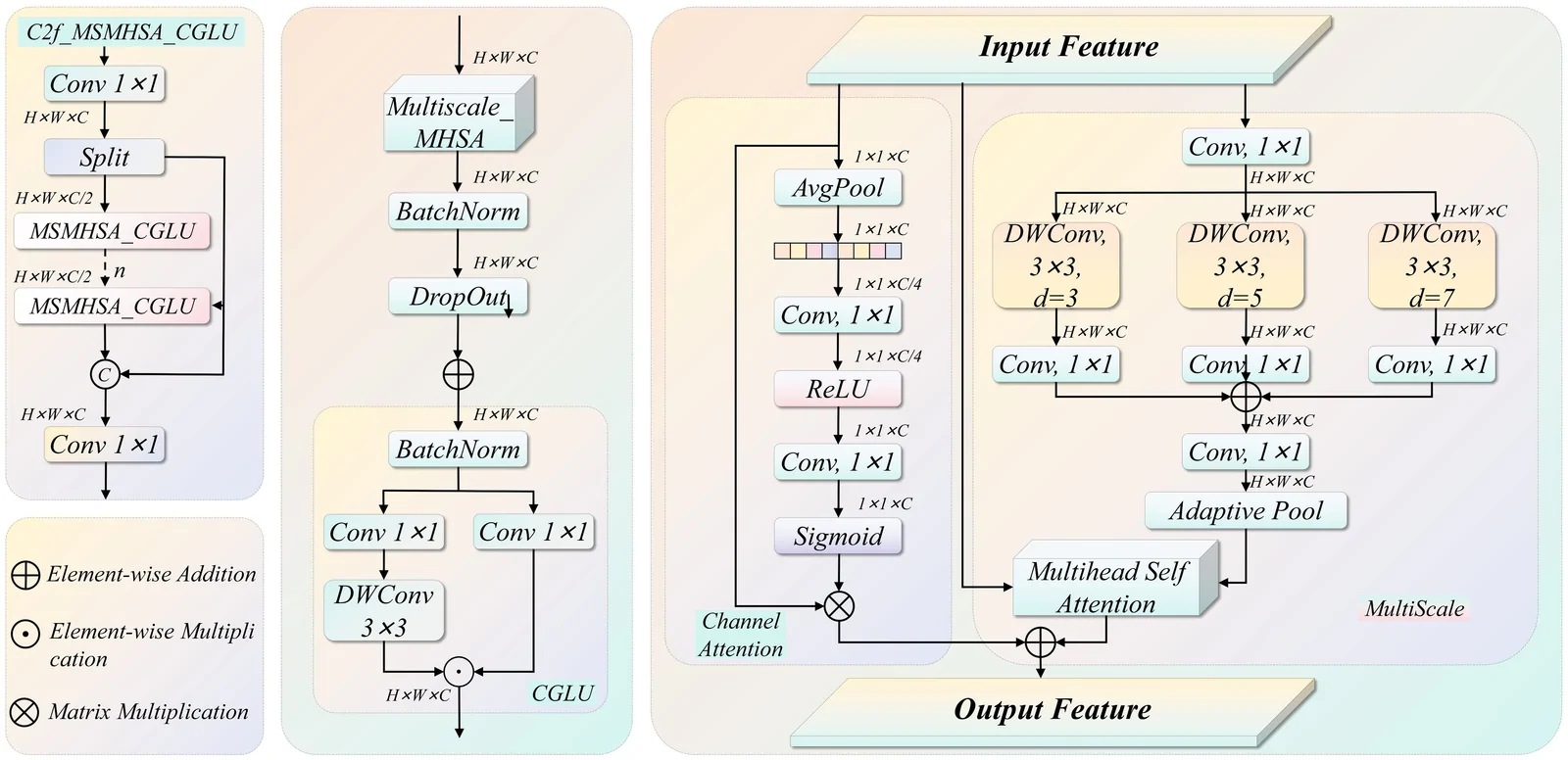
Structure of the CAG-Backbone.

CAG-Backbone adopts a five-stage hierarchical structure, in which each stage first downsamples with a 3×3 convolution of stride 2 and is then followed by a C2f_MSMHSA_CGLU module. The channel numbers of P1–P5 are 64, 128, 256, 384, and 384 in order, and their resolutions range from 1/2 to 1/32 of the input, where P3–P5 are fed into the hybrid encoder, so that shallow layers capture fine-grained local patterns and deep layers obtain semantic global representations. The feature extraction of each stage can be formalized as:

(4) $$P_{i}=H_{i}({\text{Conv}}_{↓2}(P_{i-1})),\;i=1,\ldots ,5$$

where $P_{i}\in {\mathbb{R}}^{B\times C_{i}\times \frac{H}{2^{i}}\times \frac{W}{2^{i}}}$, $H_{i}(\cdot )$ is the C2f_MSMHSA_CGLU module of the i-th stage, and ${\text{Conv}}_{↓2}$ is the downsampling convolution operator of stride 2. The overall structure of CAG-Backbone is shown in **Figure 3**.

The C2f_MSMHSA_CGLU module replaces the conventional convolutional units in the CSP bottleneck with MSMHSA_CGLU sub-modules. Given an input $X\in {\mathbb{R}}^{B\times C\times H\times W}$, after a 1×1 convolution projects it to 2c channels ( c=C·e), it is split along the channel dimension into $X_{0}$ and $X_{1}$; $X_{1}$ passes successively through n sub-module operators M(·) to generate $X_{2},\ldots ,X_{n+1}$, and all features are concatenated and restored to C channels by a 1×1 convolution:

(5) $$Y={\text{Conv}}_{1\times 1}({∥}_{k=0}^{n+1}X_{k}),\;X_{k+1}=M(X_{k})$$

Each MSMHSA_CGLU sub-module adopts a dual-branch residual structure, applying multi-scale multi-head self-attention and the convolutional GLU in turn:

(6) $$Z^{\prime }=Z+D_{p}(\text{BN}(A(Z))),\;M(Z)=Z^{\prime }+D_{p}(\text{CGLU}(Z^{\prime }))$$

where ∥ denotes the channel-wise concatenation operator, $D_{p}$ is the DropPath stochastic-depth operator, and A(·) is the multi-scale multi-head self-attention (MSMHSA). The attention-then-gating order in Equations 2–7 allows attention to first establish multi-scale spatial correspondences, after which the gated feed-forward performs channel-wise nonlinear refinement.

Multi-scale multi-head self-attention (Multiscale_MHSA) decouples the generation of the query from that of the keys and values: the query originates from the original-resolution feature map, whereas the keys and values originate from the multi-scale aggregated representation produced by the MultiScale module, so that each attention head can cover the context of a different receptive field. The attention output is fused with a parallel channel-attention branch:

(7) $$A(X)=\sigma (\frac{QK^{⊤}}{\sqrt{d_{h}}})V+\gamma (X)⊙X$$

where $Q={\text{Reshape}}_{h}(X)\in {\mathbb{R}}^{Bh\times HW\times d_{h}}$; $K,V\in {\mathbb{R}}^{Bh\times H"W"\times d_{h}}$ originate from ${\text{Conv}}_{1\times 1}(\Psi (X))$; the channel gating γ(X)=Sigmoid(FC(GAP(X))) is a squeeze-and-excitation-style bottleneck network with ReLU6 activation and Sigmoid gating; ⊙ is the Hadamard product; and σ denotes Softmax normalization as in Equations 2, 3. The multi-scale spatial branch and the channel-gating branch operate in parallel, improving the discriminability between visually similar weeds and crop patterns.

The multi-scale feature aggregation module (MultiScale) generates the multi-scale contextual representation required for the key–value projection. A shared 1×1 convolution reduces the input dimension by a compression ratio r, and three parallel depthwise separable dilated convolution branches (with dilation rates d∈{3,5,7}) extract context at different scales; the outputs of the branches are restored in channels by point-wise convolution, summed element-wise, residually modulated with the input, and finally adaptively average-pooled to P×P:

(8) $$\Psi (X)=P_{16}({\text{Conv}}_{1\times 1}(\sum_{j=1}^{3}{\text{DWConv}}_{d_{j}}(\varphi (X)))⊙X)$$

where $\varphi (X)={\text{Conv}}_{1\times 1}(X)\in {\mathbb{R}}^{B\times \frac{C}{r}\times H\times W}$ is the channel-compression projection, $d_{j}$ is the dilation rate of the j-th depthwise-convolution branch, and $P_{16}$ denotes the adaptive average-pooling operator that outputs 16×16. The dilated convolutions aggregate multi-scale context at a low computational cost, and the pooling reduces the quadratic complexity of the subsequent attention.

The convolutional gated linear unit (ConvolutionalGLU) replaces the conventional two-layer MLP with a gated architecture and incorporates depthwise separable spatial convolution. The input X is projected by a 1×1 convolution into a gating signal V and a main path U (both of dimension $\hat{C}=\lfloor 2C/3\rfloor$); U undergoes a 3×3 depthwise convolution and GELU activation and is then combined with V by a Hadamard product, and the result is projected back to the original channels by a 1×1 convolution with a global residual connection added:

(9) $$\text{CGLU}(X)=X+{\text{Conv}}_{1\times 1}(\text{GELU}({\text{DWConv}}_{3\times 3}(U))⊙V)$$

where $U,V=\text{Split}({\text{Conv}}_{1\times 1}(X))$ is the content–gating dual branch. The gating mechanism regulates the information flow through multiplicative interaction, and the local spatial inductive bias provided by the depthwise convolution complements the global modeling of the attention layer.

### 2.5 HyperFPN

**Figure 4:**
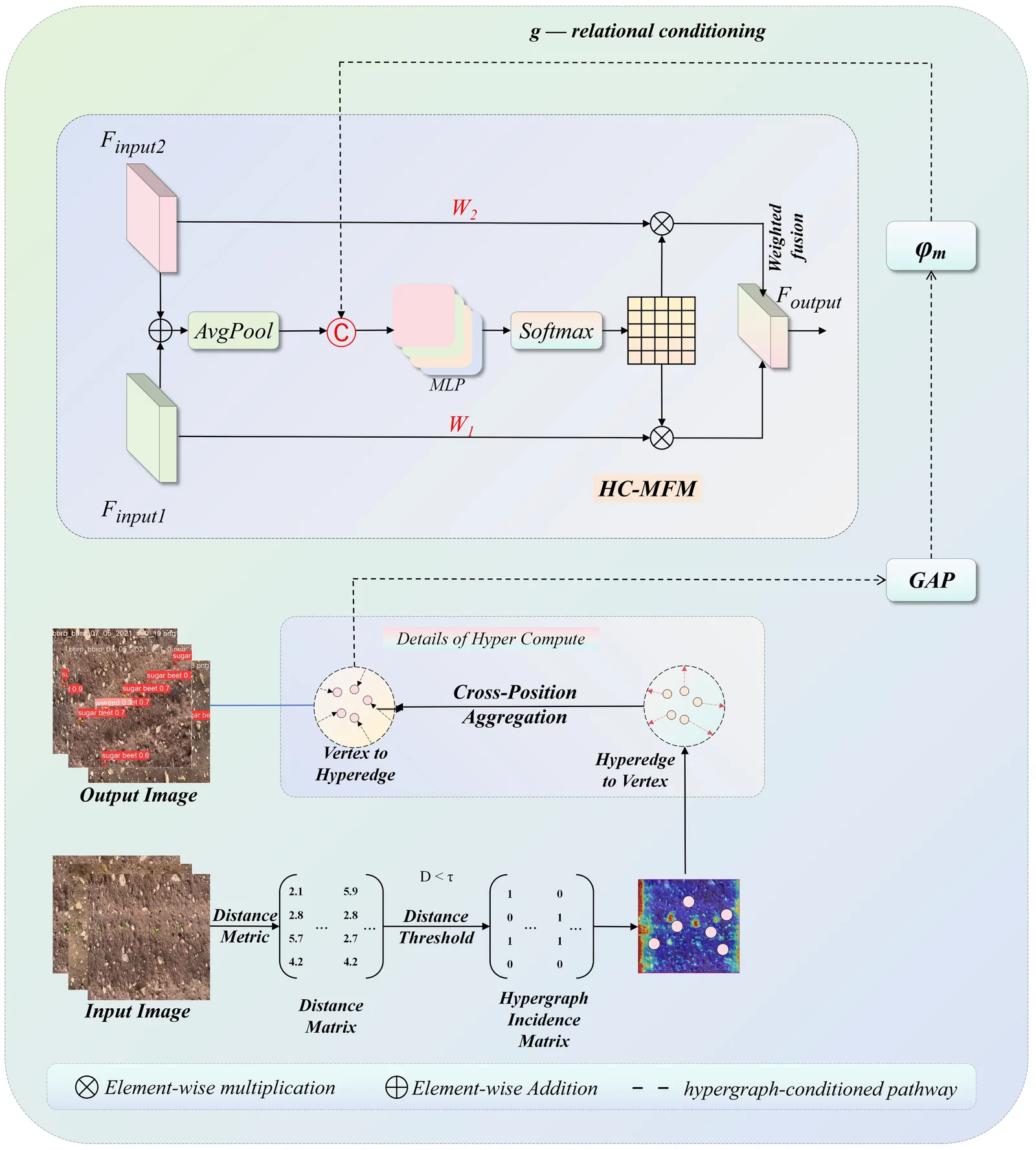
Hypergraph-conditioned multi-feature fusion (HC-MFM). The path from the hypergraph output through global average pooling and the lightweight mapping produces the group-level semantic descriptor g, which is concatenated with the pooled branch descriptor before the fusion weights are generated; removing this path reduces HC-MFM to the standard MFM of Equations 2–15 and corresponds to configuration C3 in Section 3.2. The position of HC-MFM within HyperFPN is shown in **Figure 1**, and the construction of the hypergraph is detailed in **Figure 5**. In the lower panel, node distances are computed in feature space; the field images are shown for illustration only.

**Figure 5:**
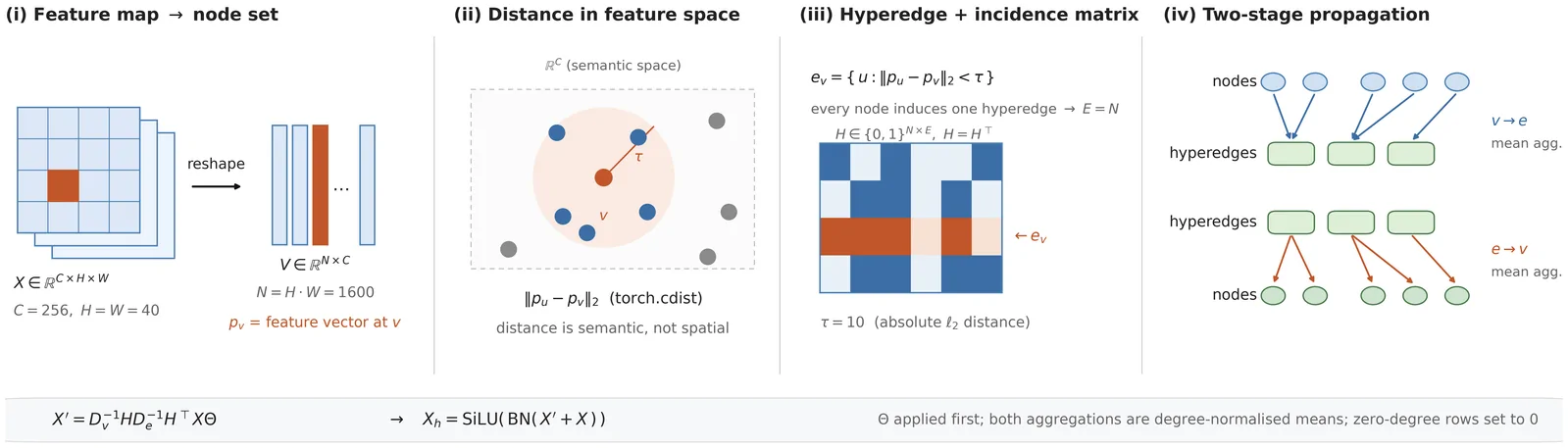
Construction and propagation of the hypergraph in HyperFPN, detailing the computation summarized in **Figure 4**. Node distances are measured in feature space rather than in image space. (i) The aggregated feature map is reshaped into N = H·W nodes, each carrying a C-dimensional feature vector. (ii) Distances between nodes are computed in feature space. (iii) Each node induces one hyperedge containing the nodes within distance τ, giving a symmetric incidence matrix with E = N. (iv) Two-stage message passing aggregates node features into hyperedges and redistributes them back, followed by a residual connection, batch normalisation and SiLU.

The conventional CCFF fuses adjacent levels by concatenation or addition. It models pairwise relationships only, expresses no high-order dependency across semantic levels, and makes no judgment about how reliable each scale is. That reliability, however, is not fixed in a sugar beet field: it shifts with object size, background texture, and cross-scale context. HyperFPN, and the HC-MFM module inside it, let hypergraph group-level semantics participate directly in generating the fusion weights. Multi-scale fusion is thereby lifted from locally-statistics-driven aggregation to feature arbitration constrained by high-order relational semantics. **Figure 4** shows the structure of HyperFPN together with the hypergraph-conditioned generation of the fusion weights inside HC-MFM, and **Figure 5** illustrates the hypergraph construction and propagation in detail.

The hypergraph construction and the HyPConv two-stage propagation follow Hyper-YOLO (Feng et al., 2025), as does the mixed-aggregation block described below; our contribution at this level lies in HC-MFM, which routes the recovered group-level semantics into the generation of the multi-scale fusion weights rather than treating the hypergraph output as an ordinary feature map. Configuration C3 in Section 3.2 tests this difference.

HyperFPN consists of three stages: semantic aggregation, hypergraph computation, and bidirectional feature propagation. Let the multi-scale features from the backbone be ${\left\{F_{i}\right\}}_{i=3}^{5}$; each level is first aligned to a unified resolution by a spatial-scale alignment operator $\Lambda_{i}(\cdot )$, and its channel dimension is unified by a 1×1 convolution, yielding the aligned features ${\tilde{F}}_{i}$:

(10) $${\tilde{F}}_{i}={\text{Conv}}_{1\times 1}(\Lambda_{i}(F_{i})),\;i\in \{3,4,5\}$$

where $\Lambda_{i}(\cdot )$ denotes the upsampling or downsampling alignment operation applied to the feature of the i-th level. The aligned multi-branch features are passed through the basic MFM (multi-feature fusion module, defined in Equations 2–15) to obtain the semantically aggregated feature $X_{h}$, which simultaneously contains shallow texture and deep semantic information and serves as the input to hypergraph computation:

(11) $$X_{h}=\text{MFM}({\tilde{F}}_{3},{\tilde{F}}_{4},{\tilde{F}}_{5})$$

After hypergraph computation, the enhanced aggregated feature ${\tilde{X}}_{h}$ is obtained. It is scattered back to the pyramid and enters the fusion stage at a single level, where it conditions the generation of the fusion weights in HC-MFM; the remaining levels are fused by the standard MFM. Each fused feature is then refined by re-parameterized convolution, generating the final multi-scale detection features $O_{i}$.

The hypergraph computation module (HyperComputeModule) jointly models the high-order semantic associations among spatial nodes by constructing a hypergraph. Given the input feature $X_{h}\in {\mathbb{R}}^{B\times C\times H\times W}$, it is first reshaped into a spatial-node sequence $V\in {\mathbb{R}}^{B\times N\times C}$, where N=H×W. A hypergraph incidence matrix $H\in {\left\{0,1\right\}}^{N\times E}$ is then constructed based on the Euclidean distance between nodes:

(12) $$H(v,e)=1(∥p_{v}-c_{e}{∥}_{2}<\tau )$$

where 1(·) is the indicator function, $p_{v}$ is the C-dimensional feature vector carried by node v, so that distances are measured in feature space rather than in image space and a hyperedge groups nodes that are semantically similar rather than merely spatially adjacent, $c_{e}$ is the centre node of the e-th hyperedge, and τ is the distance threshold controlling the connection range of the hyperedges. Hyperedges are generated from the nodes themselves: every node in turn serves as a centre and induces one hyperedge containing the nodes that lie within a distance τ of it, so that no clustering step is required and the number of hyperedges equals the number of nodes. We set τ = 10 throughout; at an input resolution of 640×640 the aggregation grid is 40×40, giving 1600 nodes at 256 channels. **Figure 5** illustrates the construction and the subsequent propagation. On the constructed hypergraph, the module performs two-stage message propagation through HyPConv (Feng et al., 2025): node-to-hyperedge aggregation first extracts high-order group-level semantics, and hyperedge-to-node back-propagation then redistributes the group-level semantics to each spatial node. This two-stage propagation follows the hypergraph convolution form of the hypergraph neural network (HGNN) (Feng et al., 2019) and can be uniformly expressed as:

(13) $$Z=D_{v}^{-1/2}HW_{e}D_{e}^{-1}H^{⊤}D_{v}^{-1/2}V\Theta$$

where Θ is the learnable weight matrix of the linear transformation, $W_{e}$ is the diagonal hyperedge-weight matrix characterizing the relative importance of each hyperedge, and $D_{e}$ and $D_{v}$ are the hyperedge-degree matrix and the node-degree matrix, respectively, which impose symmetric normalization on the propagated messages to eliminate the scale bias caused by differences in the number of node connections; when a corresponding degree is zero, it is set to zero to ensure numerical stability. Finally, the hypergraph-convolution output is post-processed by a residual connection, batch normalization, and the SiLU activation function to obtain the enhanced aggregated feature ${\tilde{X}}_{h}$.

The enhanced feature is then refined by a stack of mixed-aggregation blocks (MANet) before being scattered back to the individual scales. Each block projects the input to twice the working channel width and splits the result into four parallel parts: a pointwise branch, a depthwise branch composed of pointwise, depthwise and pointwise convolutions, and the two halves of the projected feature itself. The outputs of three sequentially applied bottlenecks are appended, and all parts are concatenated and projected back by a final pointwise convolution. We use three bottlenecks, a depthwise kernel size of 3 and an expansion factor of 2. The block follows the mixed-aggregation design of Hyper-YOLO (Feng et al., 2025) and is used here without modification.

The fusion quality of multi-branch heterogeneous features directly affects fine-grained crop–weed discrimination. Standard channel-attention fusion (i.e., the baseline MFM) typically aligns the features of each branch by 1×1 convolution, sums them element-wise, and then generates channel attention through global average pooling (GAP) and a lightweight MLP ϕ, obtaining competitive weights across the branch dimension via the Softmax operator σ. Its essence remains independent weighting, in which the fusion weights are mainly determined independently by the global statistics of each branch’s own features:

(14) $$F_{\text{out}}=\sum_{i}a_{i}⊙{\tilde{F}}_{i},\;a=\sigma (\phi (\text{GAP}(\sum_{i}{\tilde{F}}_{i})))$$

However, in scenes where crops and weeds are highly similar in appearance, the reliability of features at different scales depends on the cross-scale object–background relationship, and it is difficult to judge which branch is more reliable from each branch’s own statistics alone. HC-MFM therefore takes the enhanced aggregated feature ${\tilde{X}}_{h}$ obtained by hypergraph computation as the cross-scale group-level semantic context: it first generates a group-level semantic descriptor g through global average pooling and a lightweight mapping $\phi_{m}$, and then concatenates g with the summed descriptor of the branches to jointly generate the fusion weights:

(15) $$ɡ=\phi_{m}(\text{GAP}({\tilde{X}}_{h})),\;a=\sigma (\phi ([\text{GAP}(\sum_{i}{\tilde{F}}_{i});\;ɡ]))$$

where [· ; ·] denotes channel-wise concatenation, g is the group-level semantic descriptor from high-order hypergraph modeling, and the fusion output $F_{\text{out}}$ is still aggregated according to Equations 2–15. Compared with Equations 2–15, the key of HC-MFM lies not in simply inserting a hypergraph computation module, but in coupling hypergraph relational modeling with adaptive multi-scale fusion at the mechanism level: the fusion weights are no longer determined solely by each branch’s local statistics, but are jointly arbitrated conditioned on the cross-scale group-level semantics, thereby suppressing the contribution of unreliable scale branches and enhancing the response of discriminative scale branches in scenes where crops and weeds are similar in scale and confusable in appearance.

In Equations 2–15 and Equations 2–16, ${\tilde{F}}_{i}$ is the aligned input feature of the i-th branch, $a_{i}$ is the channel-attention weight vector corresponding to the i-th branch, ⊙ denotes element-wise multiplication in the broadcasting sense, and σ acts on the branch dimension to ensure that the weights of all branches sum to 1. The C3 ablation degrades HC-MFM to the standard MFM of Equations 2–15, retaining the multi-branch fusion structure while removing the conditional modulation of the weight generation by the hypergraph semantics g; it therefore tests whether the hypergraph semantics genuinely participate in the fusion decision. Because the conditioning pathway also carries parameters, C3 differs from the full model in parameter count as well, so the comparison is not a perfectly isolated test of the conditioning mechanism alone.

In summary, HyperFPN combines high-order hypergraph modeling with adaptive fusion: the hypergraph structure models the group-level high-order semantic dependencies among cross-scale nodes, and HC-MFM further arbitrates the fusion weights of each branch conditioned on the hypergraph group-level semantics, advancing multi-scale fusion from feature aggregation to relation-conditioned feature arbitration, thereby alleviating information redundancy and semantic mismatch and constructing a more complete multi-level feature representation.

The computational cost of hypergraph computation is governed by the number of nodes and the number of hyperedges. Because every node induces one hyperedge, the two counts are equal, and constructing the incidence matrix requires the full pairwise distance computation, whose cost grows quadratically with the node count and linearly with the channel width; each of the two propagation stages carries the same order of cost, and the linear transformation contributes a further term that is linear in the node count and quadratic in the channel width. At an input resolution of 640x640 the aggregation grid is 40x40, giving 1600 nodes at 256 channels, and the module accounts for approximately 2.07 G multiply-accumulate operations in total: about 0.66 G for the pairwise distance, 0.66 G for each of the two propagation stages, and 0.10 G for the linear map. The cost is therefore quadratic in the input area, so that doubling the input side would raise it by a factor of sixteen, and the incidence matrix itself scales with the square of the node count and with the batch size, occupying roughly 82 MB in single precision at a batch size of eight.

### 2.6 Experimental environment and hyperparameters

All experiments were conducted on a unified platform, and the hardware and software configurations are detailed in **Supplementary Table 4**.

Training follows the RT-DETR paradigm: AdamW ( $\text{lr}=1\times 10^{-4}$, $\text{wd}=1\times 10^{-4}$), with cosine annealing after a linear warm-up of 5 epochs; the batch size is 8 with 2-step gradient accumulation (an effective batch size of 16); the input resolution is 640×640; a total of 300 epochs are trained, with early stopping when the validation metric shows no improvement for 30 consecutive epochs. The loss is a weighted combination of the bipartite-matching cost, Focal classification, GIoU, and L1 regression. All compared detectors were retrained to convergence on the same training set using their official default hyperparameter settings; the comparison and full-factorial ablation experiments report point estimates from a single training run with the random seed set to 0, and only the key internal ablations in Section 3.2 and the few-shot experiments in Section 3.5 report the mean ± standard deviation. Therefore, results without multiple-seed repetition are mainly intended for relative comparison under the present fixed protocol.

### 2.7 Evaluation metrics

The model is evaluated from three aspects: accuracy, localization, and efficiency. For accuracy, we use precision P, recall R, and their harmonic mean F1; for localization, we use mAP50, mAP75, and mAP50–95 under IoU thresholds of 0.5 and 0.75 and over the COCO-style interval [0.50, 0.95], all computed by the official pycocotools; for efficiency, we use the parameter count Params (M), the computational cost GFLOPs, and the inference speed FPS. Precision, recall, and F1 are defined as follows:


$$P=\frac{TP}{TP+FP},\;R=\frac{TP}{TP+FN},\;F1=\frac{2PR}{P+R}$$

where TP, FP, and FN denote the numbers of true positives, false positives, and false negatives, respectively. For each class, a precision–recall (P–R) curve is drawn with recall as the horizontal axis and precision as the vertical axis, and the area under the curve is the average precision (AP) of that class; the mean over all classes yields the mAP:

(16) $$AP=\int_{0}^{1}P(R)\;dR,\;mAP=\frac{1}{C}\sum_{c=1}^{C}AP_{c}$$

where C is the number of classes. mAP50 and mAP75 denote the mAP computed at IoU thresholds of 0.5 and 0.75, respectively, and mAP50–95 is the average of the mAP over 10 IoU thresholds from 0.50 to 0.95 (with a step of 0.05); all three are consistent with the COCO standard. In the tables below they are abbreviated as mAP50, mAP75, and mAP50-95, respectively; FLOPs denote GFLOPs at a 640×640 input by default, and Params are given in M. For efficiency, FPS is obtained by averaging over 441 per-image inference runs on the test set on a single vGPU-32GB card with a batch size of 1 and FP32 precision; the measured coefficient of variation is approximately 1.5% ( 100.0±1.5 FPS), indicating stable speed measurement.

### 2.8 Experimental design

We select twelve detectors covering the three major paradigms as baselines. For the two-stage method, Faster R-CNN is chosen; for one-stage methods, RetinaNet, MobileNetV2-SSD, and the YOLO series YOLOv5m/v8m/v9m/v10m/v11m/v12m and YOLO26m are chosen; and for end-to-end Transformer methods, DEIM (D-FINE-M) and RT-DETR-R18—the direct baseline of this work—are chosen. All models were trained to convergence under the same protocol, a 640×640 input, and the same data split, and evaluated on the same test set.

To examine the relative level, we select four strong detectors as references: the structurally homologous RT-DETR-R18, the strong one-stage YOLO11-X, and two high-capacity real-time detection Transformers (D-FINE-L, RF-DETR-L), all trained and evaluated under the same protocol as ASSET-DETR. The parameter counts of D-FINE-L, YOLO11-X, and RF-DETR-L are approximately 1.4, 2.7, and 5.9 times that of ASSET-DETR, respectively, constituting a high-capacity reference group.

To examine the individual contributions and synergy of ASSET (A), CAG-Backbone (B), and HyperFPN (C), we take RT-DETR-R18 as the baseline (Experiment 1) and perform a full-factorial analysis of the $2^{3}=8$ combinations under a unified protocol. The three modules correspond to spatially focused encoding, cross-scale contextual representation, and relation-conditioned feature arbitration, respectively; this experiment can therefore be regarded as a decomposed validation of the three-level mechanism of this work. The computation–accuracy increments relative to the baseline and the progressive performance changes of the combinations are shown in **Supplementary Figure 4** and **S6**, respectively.

The full-factorial design examines the three modules as a whole; to further validate the key internal designs of each module, three internal ablations were carried out, based on the $2^{3}$ design. Each modifies a single component at a time on top of the full model, keeping the rest unchanged, and the modified model is retrained and re-evaluated; the three ablations target the ASSET encoder, the CAG-Backbone, and HyperFPN, respectively. Unless otherwise stated, the results are obtained under a unified data split, hyperparameters, and evaluation criteria; all configurations in these three internal-ablation tables report the mean ± standard deviation over 3 random seeds (0/1/2), with C0 and C3 serving as the key evidence for the mechanism. The results for the latter two are reported in **Supplementary Table 7** and **S8**.

The source-domain experiments evaluate performance, module contributions, and the complexity–accuracy trade-off under a fixed split. Considering that field acquisition devices, viewpoints, backgrounds, illumination, and weed composition vary with region and season, we position the external-domain experiments as a domain-shift diagnosis and a few-shot recovery analysis, centered on two questions: how much source-domain performance the model retains under the zero-shot condition, and whether a small amount of target-domain annotation can restore the detection capability. To this end, two external datasets, OD-SugarBeets and PhenoBench, are introduced, and zero-shot generalization, class-level error analysis, and few-shot adaptation experiments are conducted in turn.

Zero-shot evaluation directly infers on the external set with the source-domain weights, without using any target-domain annotation. In addition to the target-domain mAP50, we report the mean target-domain mAP, ΔmAP (Target − Source), and the retention rate (Target/Source); the few-shot setting additionally reports the gain relative to zero-shot.

## 3 Results

### 3.1 Comparison with mainstream detectors

From **Table 2**, under the present fixed split and the training protocol with random seed 0, ASSET-DETR achieves superior point estimates on all six accuracy metrics. Its mAP50–95 is 0.517, 0.6 percentage points higher than the second-best YOLO26m and 7.7 percentage points higher than Faster R-CNN; its mAP50 is 0.742, and its precision of 0.791 and recall of 0.711 also rank among the top. The per-metric distribution comparison and ranking trajectories of the detectors over the six metrics are shown in **Supplementary Figure 2** and **S3**, respectively.

**Table 2:** Comparison of ASSET-DETR with mainstream detectors on the LB sugar beet field weed dataset.

Model	P	R	F1	mAP50	mAP75	mAP50-95	FLOPs	Params	FPS
Faster R-CNN	0.730	0.666	0.697	0.690	0.480	0.440	177	44	19
RetinaNet	0.714	0.631	0.670	0.677	0.470	0.437	150	39	24
MobileNetV2-SSD	0.633	0.536	0.580	0.573	0.360	0.325	3.7	4.6	145
YOLOv5m	0.698	0.641	0.668	0.658	0.468	0.436	49.0	21.2	116
YOLOv8m	0.764	0.687	0.723	0.721	0.528	0.481	78.9	25.9	93
YOLOv9m	0.761	0.684	0.720	0.714	0.536	0.501	76.3	20.0	82
YOLOv10m	0.766	0.685	0.723	0.722	0.533	0.494	59.1	15.4	108
YOLOv11m	0.777	0.697	0.735	0.727	0.541	0.503	68.0	20.1	99
YOLOv12m	0.780	0.700	0.738	0.730	0.544	0.507	67.5	20.2	89
YOLO26m	0.784	0.704	0.742	0.734	0.547	0.511	68.2	20.4	102
DEIM (D-FINE-M)	0.781	0.699	0.738	0.731	0.545	0.509	57.0	19.2	94
RT-DETR-R18	0.728	0.649	0.686	0.664	0.470	0.440	42.3	15.52	124
**ASSET-DETR (Ours)**	**0.791**	**0.711**	**0.749**	**0.742**	**0.550**	**0.517**	**48.6**	**21.34**	**100**

The values are point-estimate results on the test set from a single training run (random seed 0); the meanings of mAP50, mAP75, mAP50-95, FLOPs, and Params are the same as in Section 2.7.

Bold values indicate the results of the proposed ASSET-DETR model.

**Figure 6:**
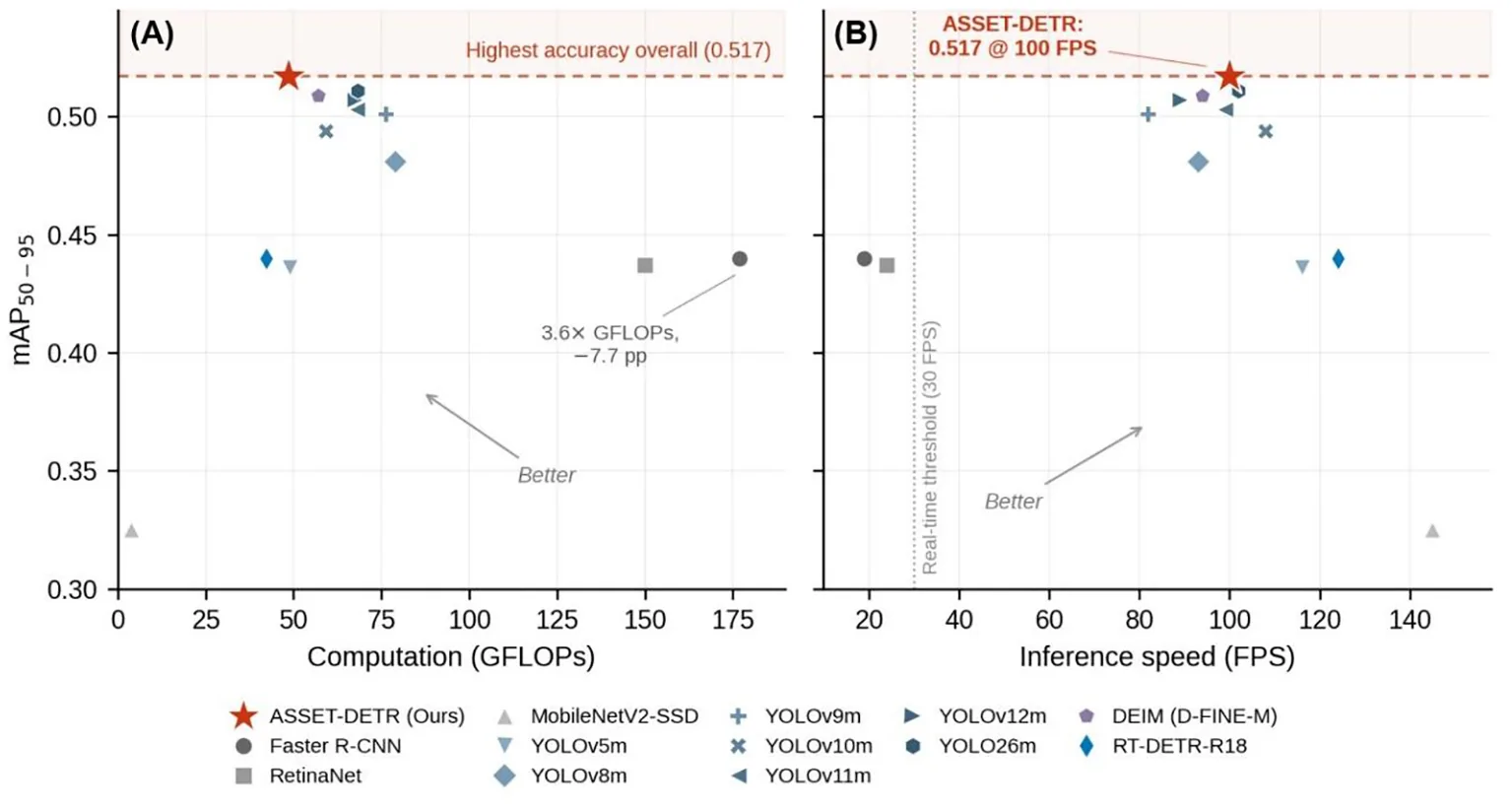
Accuracy–efficiency trade-off between mAP50–95 and **(A)** computational cost and **(B)** inference speed for the detectors.

In terms of efficiency (**Figure 6**), ASSET-DETR attains an mAP50 of 0.742 and an mAP50–95 of 0.517 at 100 FPS (10.0 ms/frame), forming a favorable accuracy–efficiency trade-off among the compared models; no model simultaneously achieves better point estimates on both dimensions. Relative to RT-DETR-R18, mAP50 and mAP50–95 improve by 7.8 and 7.7 percentage points, respectively, while the parameter count increases from 15.52 M to 21.34 M (an increase of 37.5%), GFLOPs increase from 42.3 to 48.6, and FPS decreases from 124 to 100.

Compared with the YOLO series, the FPS of ASSET-DETR is slightly lower than that of YOLOv5m and YOLO26m and roughly on par with YOLOv11m, but its mAP50–95 is higher by 8.1, 0.6, and 1.4 percentage points, respectively, and 100 FPS still far exceeds the real-time threshold of about 30 FPS for field operations.

### 3.2 Ablation results

#### 3.2.1 Full-factorial ablation of the three modules

The results of the eight configurations are reported in **Table 3**. From Experiments 2–4, introducing each of the three modules individually yields positive gains. CAG-Backbone (Exp. 3) yields the largest single-module gain, raising mAP50 by 3.5 percentage points; ASSET (Exp. 2) improves mAP50 by 2.6 percentage points; and HyperFPN (Exp. 4) improves mAP50 by 2.7 percentage points and recall by 2.4 percentage points.

**Table 3:** Ablation results of the three core modules (Experiment 1 is the RT-DETR-R18 baseline).

No.	A	B	C	P	R	F1	mAP50	mAP75	mAP50-95	FLOPs	Params
Exp. 1	—	—	—	0.728	0.649	0.686	0.664	0.470	0.440	42.3	15.52
Exp. 2	✓	—	—	0.745	0.662	0.701	0.690	0.480	0.460	44.1	16.92
Exp. 3	—	✓	—	0.753	0.669	0.709	0.699	0.500	0.471	45.5	17.82
Exp. 4	—	—	✓	0.742	0.673	0.706	0.691	0.490	0.465	44.1	17.64
Exp. 5	✓	✓	—	0.761	0.682	0.719	0.711	0.530	0.491	46.2	19.22
Exp. 6	✓	—	✓	0.760	0.679	0.717	0.710	0.510	0.480	46.0	19.20
Exp. 7	—	✓	✓	0.755	0.673	0.712	0.707	0.530	0.500	47.7	19.94
**Exp. 8**	**✓**	**✓**	**✓**	**0.791**	**0.711**	**0.749**	**0.742**	**0.550**	**0.517**	**48.6**	**21.34**

Bold values indicate the results of the complete ASSET-DETR model (Exp. 8).

Combining any two modules (Exps. 5–7) brings an mAP50 improvement of approximately 4.3–4.7 percentage points over the baseline. Measured by a synergy coefficient, the combination of A and C is the highest at about 0.87, followed by A and B at about 0.77, and B and C the lowest at about 0.69; all three are slightly below 1.

The full model (Exp. 8) attains an mAP50 of 0.742 and an mAP50–95 of 0.517, improving by 7.8 and 7.7 percentage points over the baseline (Exp. 1), by a further 3.1 percentage points in mAP50 over the best two-module combination in mAP50 (Exp. 5), and by 1.7 percentage points in mAP50–95 over the best two-module combination in mAP50-95 (Exp. 7).

In terms of the efficiency cost, from Exp. 1 to Exp. 8 the parameter count increases from 15.52 M to 21.34 M (an increase of 37.5%), GFLOPs increase from 42.3 to 48.6 (an increase of 14.9%), and FPS decreases from 124 to 100. The two-module configurations Exp. 5 and Exp. 7 carry 19.22 M and 19.94 M parameters and reach mAP50 of 0.711 and 0.707, respectively.

#### 3.2.2 Internal ablation of the ASSET encoder

**Table 4** reports the internal ablation of the ASSET encoder. Removing either branch of ASSA degrades accuracy, but not symmetrically. Retaining only the dense Softmax branch (A1) costs 1.3 percentage points of mAP50, whereas retaining only the sparse ReLU² branch (A2) costs 2.0 percentage points. Under the present protocol, removing the dense branch is therefore the more damaging of the two, indicating that preserving global context carries the larger marginal contribution to overall detection accuracy on this benchmark. The sparse branch nevertheless yields a non-redundant gain, since A1 also falls short of the full model: sparse gating contributes background suppression and selective enhancement that the dense branch does not supply on its own. The two branches are thus complementary rather than substitutable, and their adaptive fusion outperforms either single branch. Degrading SEFN to a standard FFN (A3) reduces mAP50 by 1.7 percentage points, validating the effectiveness of its spatially enhanced feed-forward structure in preserving local spatial structure.

**Table 4:** Internal ablation of the ASSET encoder.

Configuration	mAP50 (%)	mAP50-95 (%)	Params
A0 Full	74.2 ± 0.3	51.7 ± 0.4	21.34
A1 Softmax only	72.9 ± 0.3*	50.2 ± 0.3*	20.1
A2 ReLU² only	72.2 ± 0.3*	49.6 ± 0.4*	20.1
A3 Standard FFN	72.5 ± 0.3*	49.9 ± 0.3*	19.9

*Indicates that the paired t-test difference between the variant and the full model is significant (p < 0.05); Params are given under a unified statistical criterion, in units of M.

#### 3.2.3 Internal ablation of the CAG-backbone

Degrading the multi-scale dilation to a single receptive field (B1) brings the largest drop, reducing mAP50 by 2.3 percentage points, indicating that multi-scale multi-head self-attention is crucial for modeling cross-scale seedling–weed relationships; switching to the {2,4,6} combination (B2) is slightly lower than {3,5,7} by 0.5 percentage points, supporting our choice of the dilation-rate combination. Removing the CGLU gating (B3) reduces mAP50 by 1.3 percentage points, indicating that the convolutional gated linear unit helps strengthen the selective expression across channels.

#### 3.2.4 Internal ablation of HyperFPN

In these internal-ablation results (**Supplementary Table 8**), C2 does not reach significance in mAP50 (p = 0.058), confirming the robustness of HyperFPN to the hyperedge threshold τ.

The hyperedge threshold τ has a mild effect on performance within the examined range (C0 uses τ = 10, while C1 and C2 use a smaller and a larger threshold than C0, respectively; C1 and C2 differ from C0 by no more than 0.6 percentage points), indicating that HyperFPN is insensitive to this hyperparameter and has good robustness. More importantly, C3 retains the multi-branch fusion structure while degrading HC-MFM to the standard MFM of Equations 2–15, i.e., removing the conditional modulation of the fusion weights by the hypergraph semantics g. This comparison is used to test the factor of whether the hypergraph relational semantics genuinely participate in the fusion decision. The results show that mAP50 drops from 74.2 ± 0.3 to 73.5 ± 0.3 and mAP50–95 drops from 51.7 ± 0.4 to 50.9 ± 0.3 (in %; paired t-test, p < 0.05), while the parameter count decreases from 21.34 M to 20.15 M.

### 3.3 Attention-focus visualization

**Figure 7:**
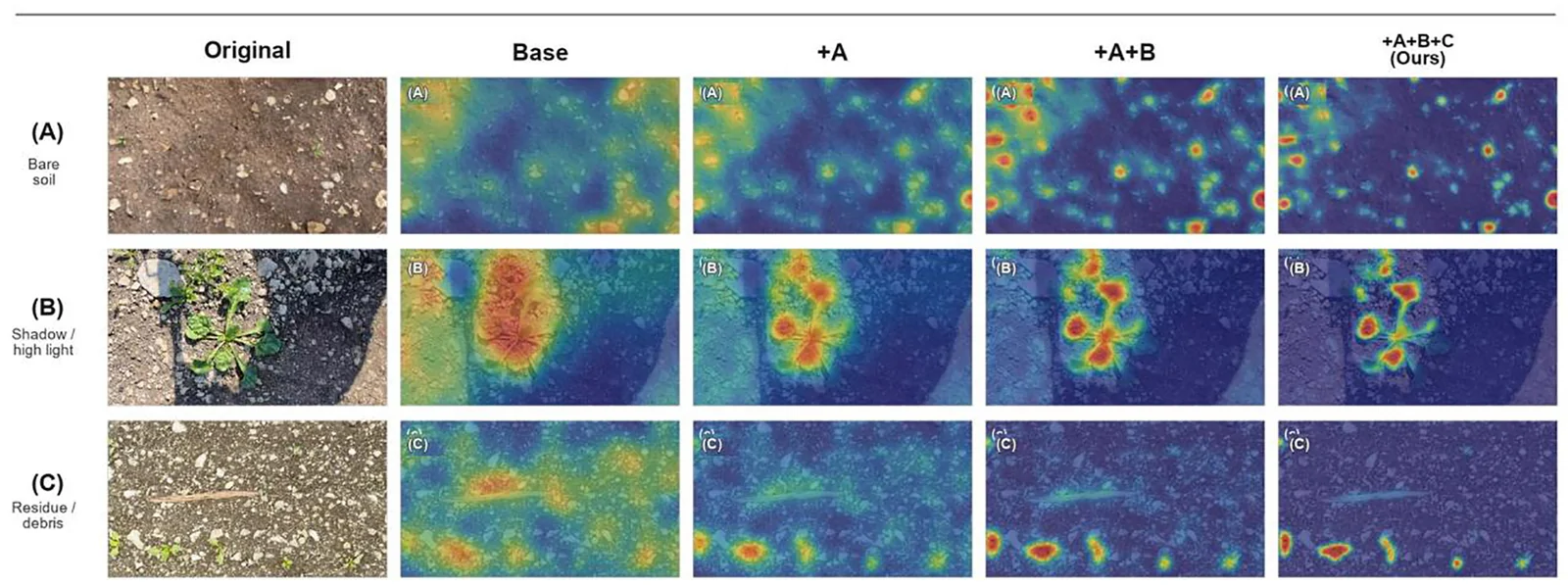
Grad-CAM++ response comparison of the ablation configurations under three typical field backgrounds (A: ASSET; B: CAG-Backbone; C: HyperFPN; row **(A)** bare-soil background, row **(B)** shadow and strong light, row **(C)** straw-residue interference).

Grad-CAM++ is applied at the P5 layer to compare class activations for the baseline and the ablation configurations that progressively introduce the three modules (baseline, +A, +A+B, +A+B+C, where A, B, and C correspond to ASSET, CAG-Backbone, and HyperFPN, respectively), covering three typical field backgrounds—bare soil, shadow and strong light, and straw residue (**Figure 7**). In all three scenes, the responses of the baseline RT-DETR-R18 are generally diffuse, with many activations falling on background regions such as bare soil, stones, shadows, and withered-leaf residue; after ASSET is introduced, background responses are markedly suppressed and the activations begin to contract toward the object regions, consistent with the design in which ReLU² sparse gating suppresses background responses. After training, ASSA converges to a state in which the sparse branch carries the larger weight ( $w_{1}/w_{2}\approx 1.8$ at random seed 0), indicating that background suppression is actively exploited in this scene type. This weighting does not, however, make the sparse branch the more indispensable of the two: as configuration A2 in Section 3.2 shows, removing the dense branch entirely costs more accuracy than removing the sparse one, so the two are complementary rather than substitutable; after CAG-Backbone is further introduced, the responses refine from coarse blobs at the whole-plant scale into discrete hotspots at the leaf and even seedling-individual scale, reflecting the contribution of multi-scale context modeling to fine-grained spatial localization; the full model highly concentrates the activation energy on the object body under all three backgrounds, with shadow boundaries and residue-interference regions producing almost no response, and even very small seedlings scattered in bare soil forming clear, mutually separated activation peaks.

### 3.4 Failure-case analysis

An attribution of the 47 failure samples with IoU below 0.5 on the test set divides them into three categories. A common characteristic across all three is that the model does respond at the correct location in most cases: the failures arise predominantly from imprecise localization rather than from a complete absence of detection, so the predicted box is displaced or poorly scaled relative to the annotation rather than missing altogether. The largest share is the extreme-occlusion type, accounting for about 38.3%; in such samples, morphologically similar broad-leaved weeds fully overlap with sugar beet leaves and the veins are invisible, so that the boundary between the weed and the crop cannot be resolved and the predicted box is drawn across both, resulting in near-neighbor box confusion. Next is the very-small-object type, accounting for about 31.9%; when an object is smaller than 24×24 pixels, the effective features are insufficient, and although the object is usually localized approximately, the predicted extent is unreliable at this scale. In addition, there is the canopy-occlusion type, accounting for about 29.8%; here the weed is occluded by more than half by the mature-stage sugar beet canopy, so that only fragments of the target remain visible and the predicted box tends to enclose the visible fragment rather than the annotated instance; such training samples account for less than 1.2% of the training set.

**Figure 8:**
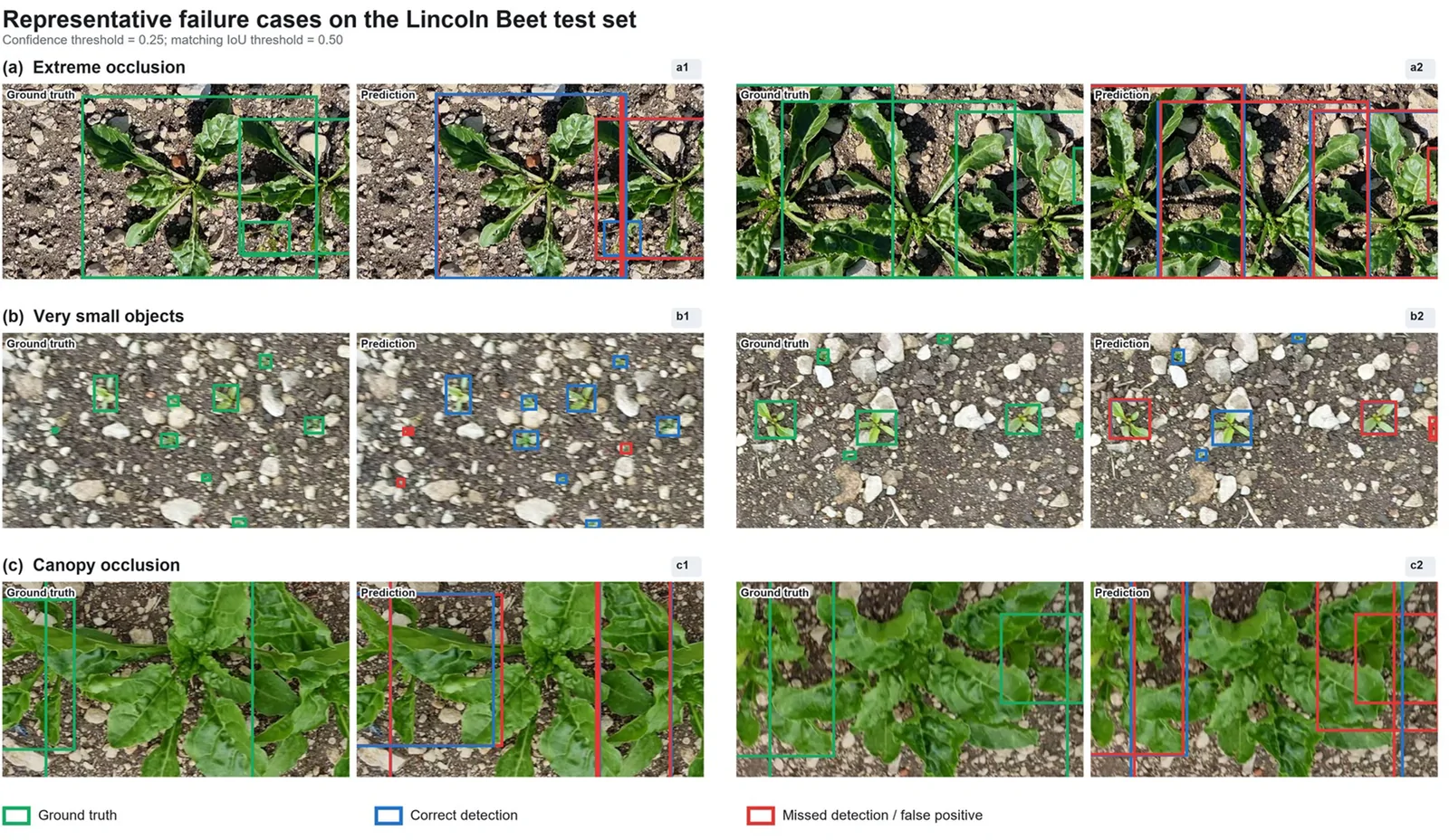
Representative failure cases of ASSET-DETR on the Lincoln Beet test set. Each pair shows the ground truth (left) and the prediction at a confidence threshold of 0.25 (right). Rows correspond to the three failure categories identified above: **(a)** extreme occlusion, in which morphologically similar broad-leaved weeds overlap with sugar beet leaves and the predicted box is drawn across both; **(b)** very small objects, for which the target is localized approximately but the predicted extent is unreliable; and **(c)** canopy occlusion, in which only fragments of the weed remain visible beneath the mature-stage canopy. Green boxes denote ground truth, blue boxes correct detections (IoU ≥ 0.5), and red boxes missed detections and false positives.

Representative cases of each category are shown in **Figure 8**.

### 3.5 Cross-domain and few-shot adaptation results

#### 3.5.1 Zero-shot generalization of ASSET-DETR

Under the zero-shot condition, ASSET-DETR exhibits a clear cross-domain drop: the source-domain mAP50 is 0.742, which decreases to 0.374 and 0.452 on OD-SugarBeets and PhenoBench, respectively, with a mean target-domain value of 0.413, a decrease of 0.329 relative to the source domain and a retention rate of approximately 55.7%, quantifying the impact of domain shift. This result indicates that the model still degrades markedly on unseen external domains, rather than having already acquired sufficient cross-domain robustness.

Among them, PhenoBench (top-view, with relatively regular imaging) is better retained than OD-SugarBeets, the latter degrading more markedly because of larger differences in weed morphology and scale; this inter-dataset difference is further localized to specific classes in the subsequent class-level analysis.

#### 3.5.2 Class-level zero-shot results

**Figure 9:**
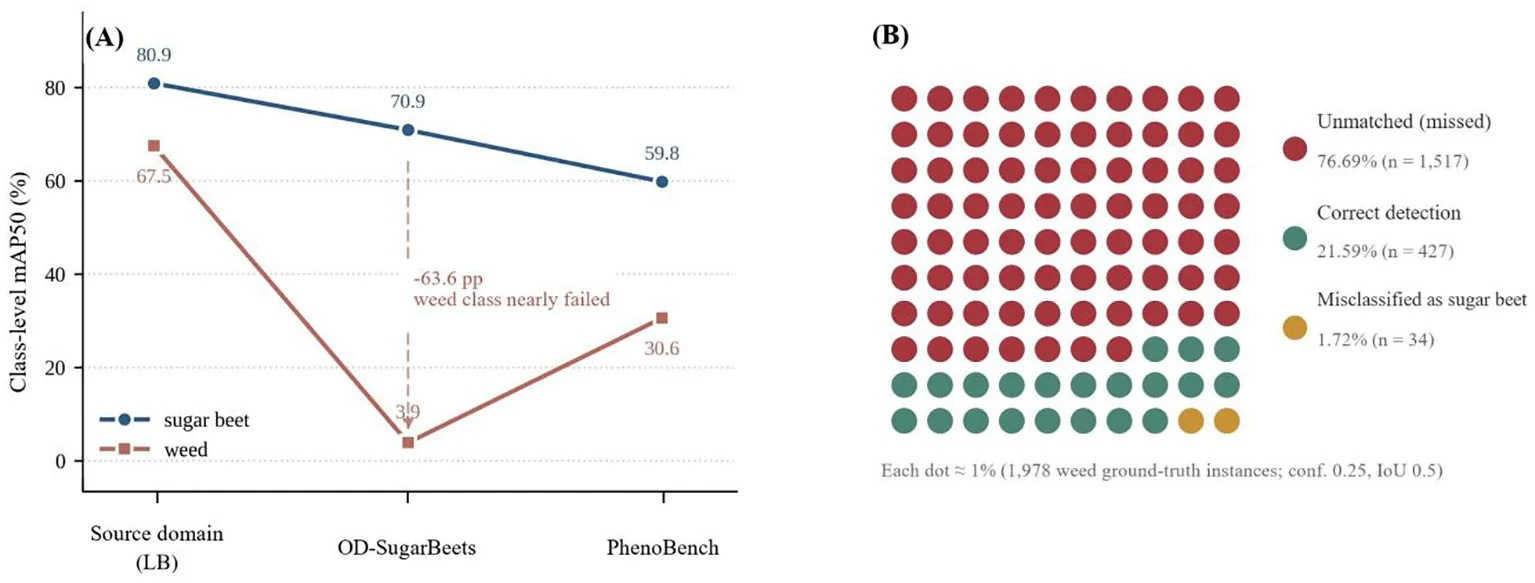
Class-level analysis of zero-shot cross-domain degradation: **(A)** mAP50 of the two classes on the source domain and the two external domains; **(B)** the matching-diagnosis composition of the weed-class ground-truth instances on OD-SugarBeets (confidence 0.25, IoU 0.5).

The class-level analysis shows that on OD-SugarBeets, mAP50/mAP50–95 is 0.374/0.210, a decrease of 0.368/0.307 relative to the source domain; the sugar beet class drops from 0.809 to 0.709 (−0.100), whereas the weed class drops from 0.675 to 0.039 (−0.636). That is, the overall loss is driven mainly by the failure of the weed class rather than by a proportional decline in both classes; the comparison of the two classes over the three domains is shown in **Figure 9(A)**.

A greedy one-to-one matching diagnosis is performed on the 1,978 weed ground-truth instances in all 964 test images of OD-SugarBeets at a confidence of 0.25 and an IoU of 0.5: correct detections number 427 (21.59%), those misclassified as sugar beet number only 34 (1.72%), and unmatched ones number 1,517 (76.69%); the error composition is shown in **Figure 9(B)**. It can be seen that the zero-shot degradation of the weed class is dominated by object localization/discovery failure and large-scale non-matching, with very little class confusion, and the bottleneck is closer to object-discovery failure caused by cross-domain differences in appearance, scale, and imaging. Typical cases are shown in **Supplementary Figure 5**.

Although the model produces many predicted responses for weeds, the IoU between the predicted boxes and the ground truth mostly fails to reach 0.5, manifesting overall as large-scale non-matching (missed detection) with only very few misclassified as sugar beet. On PhenoBench, mAP50/mAP50–95 is 0.452/0.328, and the mAP50 of the sugar beet and weed classes is 0.598 and 0.306, respectively. Compared with OD-SugarBeets, its sugar beet class is 0.111 lower and its weed class 0.267 higher, indicating that the two target domains differ not only in overall difficulty but also in class-related shift structure; the specific impact still needs to be verified in combination with object-size statistics and qualitative examples, and the results are shown in **Table 5**.

**Table 5:** Zero-shot generalization results of ASSET-DETR on the external datasets.

Dataset	Protocol	Target-domain training images	P	R	F1	mAP50	mAP50-95	mAP50 drop
Source test	In-domain	—	0.791	0.711	0.749	0.742	0.517	0.000
OD-SugarBeets	Zero-shot	0	0.499	0.383	0.433	0.374	0.210	0.368
PhenoBench	Zero-shot	0	0.581	0.495	0.534	0.452	0.328	0.290

The drop values are computed with the source test as reference.

#### 3.5.3 Few-shot target-domain adaptation

**Figure 10:**
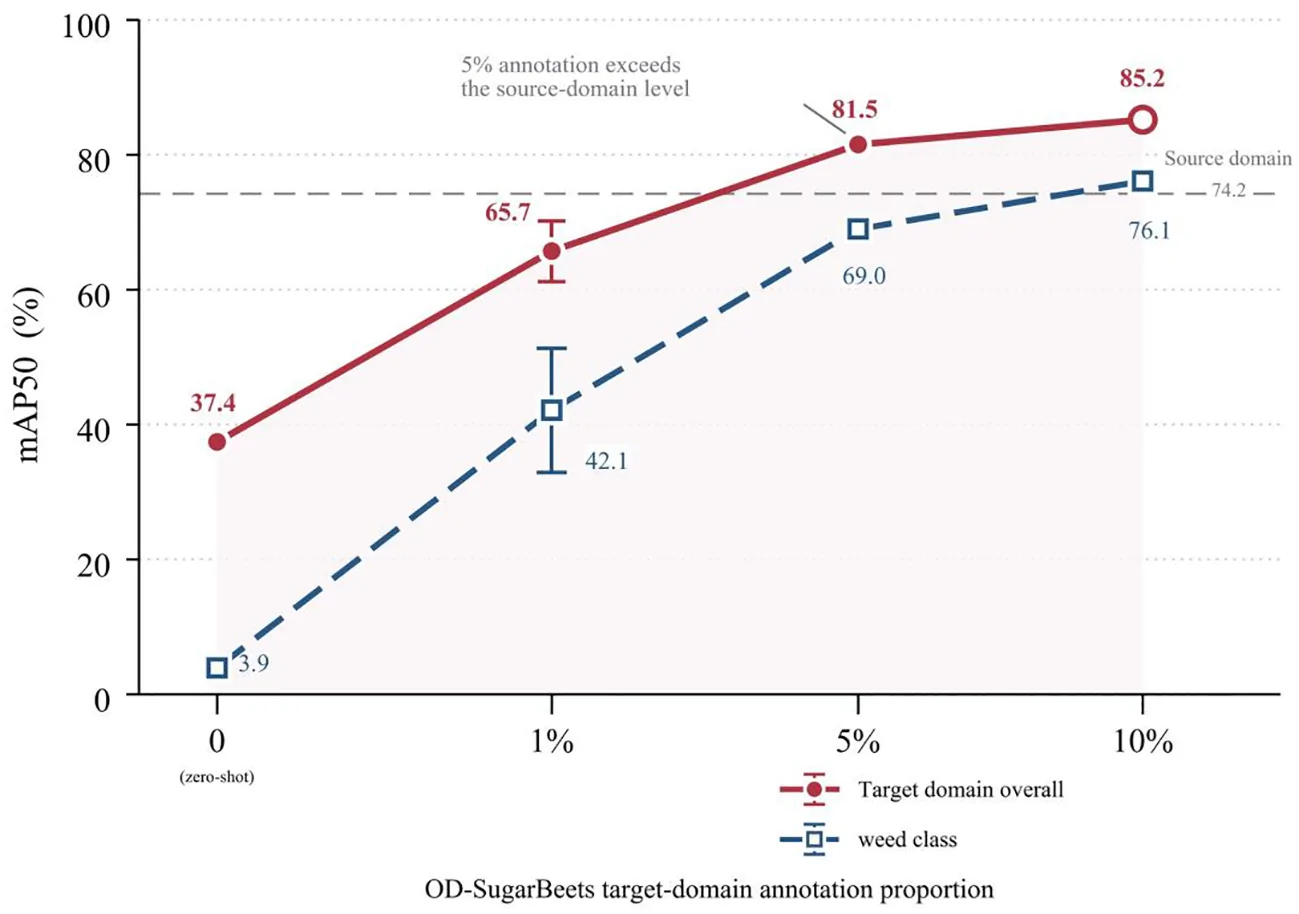
Few-shot adaptation curve on the OD-SugarBeets target domain (error bars are the standard deviation over 3 support sets; hollow points are the point estimates of a single support set; the dashed line is the source-domain mAP50 reference).

For the strong domain shift of OD-SugarBeets, we further examine the performance as the proportion of target-domain annotation increases: when fine-tuning with only 1% of the training images (35 images, 115 boxes), the target-domain mAP50, based on 3 support sets, rises from the zero-shot 0.374 to 0.657 ± 0.046, and the weed-class mAP50 rises from 0.039 to 0.421 ± 0.092 (**Figure 10**). This result indicates that a small amount of target-domain supervision can markedly alleviate the object-discovery failure under the present protocol.

When the annotation increases to 5%, the target-domain mAP50/mAP50–95 further reaches 0.815 ± 0.009/0.480 ± 0.004, and the weed-class mAP50 reaches 0.690 ± 0.021; under the 10% setting (single support set), they reach 0.852/0.501, respectively.

As shown in **Figure 10** and **Supplementary Table 1**, as the annotation proportion increases from 1% to 10%, the target-domain mAP50 rises from the zero-shot 0.374 to 0.657/0.815/0.852, and the most severely affected weed class rises from 0.039 to 0.761, indicating that, under the present setting, a small amount of target-domain annotation and fine-tuning helps alleviate the cross-domain degradation on this target domain.

#### 3.5.4 Cross-dataset comparison with strong baseline detectors

Note: Params and GFLOPs are counted for each model at a 640×640 input; FPS is measured on the same NVIDIA vGPU-32GB platform with FP32 precision and a batch size of 1.

**Figure 11:**
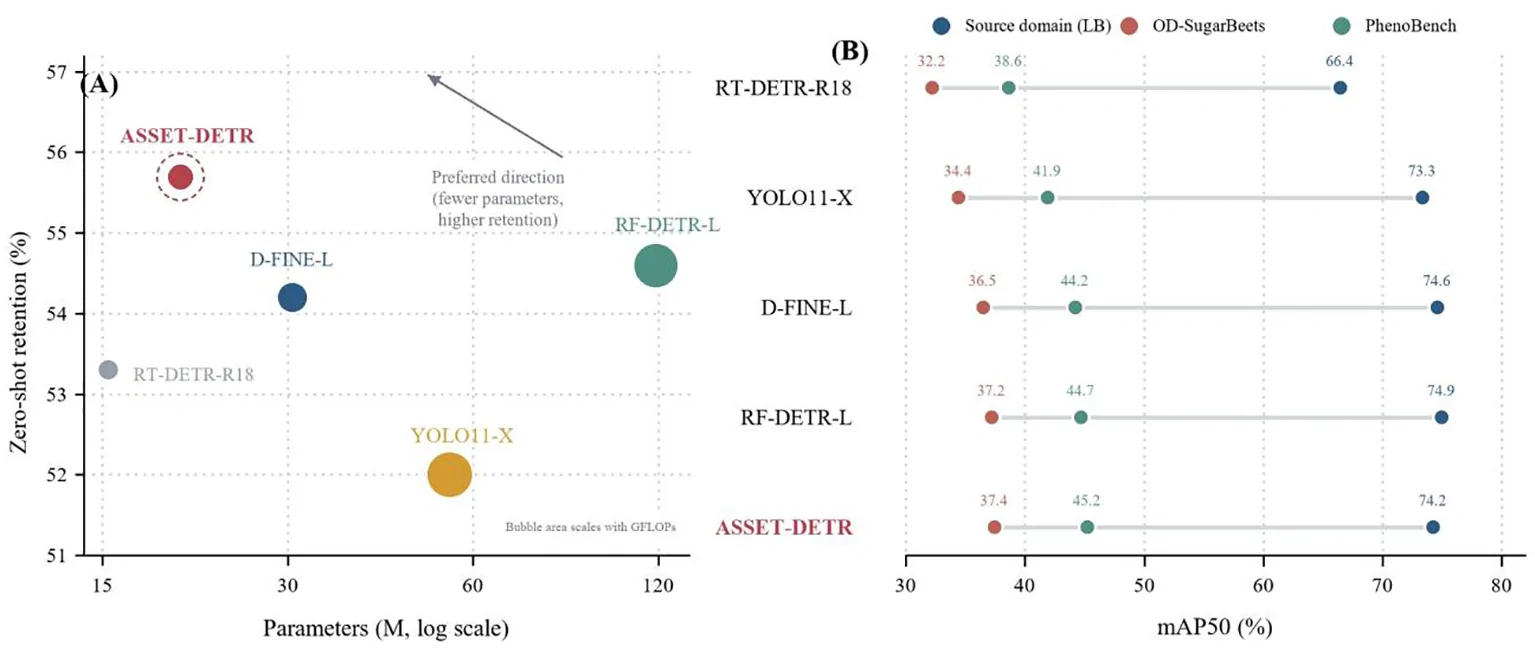
Cross-dataset zero-shot generalization comparison of the five detectors: **(A)** the relationship between the zero-shot retention rate and the parameter count (bubble area is proportional to GFLOPs); **(B)** the mAP50 of each model on the source domain and the two external domains.

Note: The mean target-domain value is the arithmetic mean of the mAP50 on OD-SugarBeets and PhenoBench; ΔmAP is the difference between the mean target-domain value and the source-domain performance; the retention rate is the ratio of the mean target-domain value to the source-domain performance.

The source-domain performance and complexity of the five models are compared in **Table 6**, and their zero-shot generalization is shown in **Supplementary Table 6** and **Figure 11**. All models exhibit a clear cross-domain drop, with retention rates ranging from 52.0% to 55.7%, indicating that domain shift is a common challenge for this task. ASSET-DETR achieves the highest point estimate under the present protocol with a retention rate of 55.7%, higher than RF-DETR-L, D-FINE-L, and RT-DETR-R18, with YOLO11-X the lowest. It attains the highest mean target-domain value and retention rate while using about 17% of the parameters of RF-DETR-L and 37% of those of YOLO11-X, indicating that the proposed relation-aware fusion mechanism has a certain parameter-efficiency advantage under the present external-domain setting.

**Table 6:** Source-domain performance and complexity comparison of the strong baseline detectors and ASSET-DETR.

Model	Source mAP50	Params	FLOPs@640	FPS
RT-DETR-R18	0.664	15.52	42.3	124
ASSET-DETR	0.742	21.34	48.6	100
D-FINE-L	0.746	30.7	91.8	65
YOLO11-X	0.733	57.1	192.1	47
RF-DETR-L	0.749	126.3	166.4	33

Beyond the zero-shot setting, we further examine label-efficient adaptation: **Supplementary Table 2** compares the few-shot adaptation under 5% annotation, showing that a small amount of annotation substantially restores the mAP50 of each model on OD-SugarBeets, with retention rates returning to 98.5%–109.8%. ASSET-DETR achieves the largest gain and the highest point-estimate retention rate under the present 5% support setting, outperforming RF-DETR-L, D-FINE-L, and YOLO11-X. This result supports the view that its utilization of a small amount of in-field annotation is relatively effective under the present external-domain setting, but more support sampling and repetitions over optimization randomness are still needed to assess stability.

## 4 Discussion

### 4.1 Contribution and synergy of the three mechanisms

The full-factorial results admit a consistent mechanistic reading. The largest single-module gain comes from CAG-Backbone, which is consistent with its design in which multi-scale long-range modeling alleviates the single-scale limitation of ResNet; the gain of ASSET corresponds to the noise suppression of ASSA and the spatial prior of SEFN; and the gain of HyperFPN, which is largest in recall, is consistent with high-order semantic modeling and relation-conditioned fusion improving the recovery of small and ambiguous objects. That the full model exceeds every two-module combination indicates that the three mechanisms are complementary under the present protocol, while their common orientation toward object focus and scale discrimination under complex backgrounds implies a degree of functional overlap.

The synergy coefficients quantify that overlap. All three pairwise coefficients fall slightly below 1, showing mild sub-additivity: CAG-Backbone and HyperFPN overlap most notably in multi-scale discrimination, whereas ASSET is the more complementary of the three. The accuracy gain is therefore not attributable to added capacity alone. The two-module configurations Exp. 5 and Exp. 7 already carry 19.22 M and 19.94 M parameters, so the final 1.4 to 2.1 M parameters contributed by the third module yield a further 3.1 to 3.5 percentage points of mAP50 that the intermediate configurations do not deliver. At 21.34 M parameters ASSET-DETR also remains substantially smaller than the strongest cross-domain baselines examined in Section 3.5, while retaining a higher proportion of its source-domain accuracy on both external datasets; combined with the real-time inference speed of 100 FPS, this complexity cost remains within the acceptable range for real-time field detection.

The overlap between CAG-Backbone and HyperFPN has a concrete structural explanation. The two components aggregate multi-scale context at different stages: the multi-scale branch inside MSMHSA constructs a dilated multi-receptive-field representation within each backbone stage, whereas HyperFPN establishes group-level relations across the aligned pyramid levels after the encoder, so that part of the multi-scale evidence is constructed twice. This suggests a concrete route to reducing the parameter overhead that we have not yet implemented: letting the hypergraph stage reuse the multi-scale key-value aggregation already computed in the backbone, rather than recomputing cross-scale context in the neck. Restricting hypergraph-conditioned fusion to the coarser pyramid levels, and sharing the query-key-value projection across attention heads, offer two further reductions. Disentangling the two components in this way, and verifying that accuracy is preserved at a lower parameter budget, is left to future work.

Because configuration C3 differs from the full model in parameter count as well as in the conditioning pathway, this comparison does not isolate semantic conditioning from model capacity completely. The observed drop is nevertheless disproportionate to the 5.6% reduction in parameters, and is consistent with the high-order relational semantics imposing an effective constraint on scale-weight arbitration rather than the gain arising from parameter scale alone.

Several further observations point in the same direction. The gains over RT-DETR-R18 are attributable to the reconstruction of the backbone representation by CAG-Backbone, and to ASSET and HyperFPN suppressing background redundancy and strengthening high-order semantic association, respectively; given that sugar beet seedlings and accompanying weeds have similar leaf shapes, the resulting accuracy is consistent with the design goal of the three modules to enhance fine-grained crop–weed discrimination. The Grad-CAM++ comparison in Section 3.3 corroborates this at the level of spatial response: the effects of the three modules on attention focus are progressive and complementary, providing mechanism-level evidence alongside the quantitative ablation results of Section 3.2.

### 4.2 Comparison with previous methods

Placing these results against previous work clarifies both what the proposed design inherits and where it departs. At the attention level, the sparsification strategies that motivate ASSA differ in how they restrict the attention field: Swin Transformer (Liu et al., 2021) confines attention to shifted local windows, Sparse DETR (Roh et al., 2022) updates only salient tokens, and SparseFormer (Gao et al., 2024) recognizes a scene from a small budget of latent tokens. Each imposes sparsity as a fixed structural constraint. ASSA instead follows the adaptive sparse–dense formulation of Zhou et al. (2024), in which the balance between the two branches is learned rather than prescribed. The ablation in Section 3.2 supports the value of keeping both branches, and also qualifies a common assumption in this line of work: removing the dense branch is more damaging than removing the sparse one, so sparsification is best understood here as a complement to global context rather than a replacement for it. On the feed-forward side, SEFN follows CvT (Wu et al., 2021) and TransNeXt (Shi, 2024) in injecting convolutional spatial priors, and the A3 configuration confirms that this matters for crop–weed discrimination.

At the fusion level the departure is larger. FPN (Lin et al., 2017a) established the top-down paradigm, AFPN (Yang et al., 2023) narrows the semantic gap between non-adjacent levels through progressive fusion, and MSE-FPN (Guo et al., 2023a) adds multi-scale semantic enhancement; Fog-UAVNet (Dong et al., 2026) adapts the multi-scale prior to degraded imaging conditions. All of these remain grounded in pairwise relationships between levels, and none forms an explicit judgment of how reliable each scale is in the current scene. The hypergraph literature supplies the missing relational primitive but has not been used in this role: in HGNN (Feng et al., 2019), HGNN+ (Gao et al., 2023), Hyper-YOLO (Feng et al., 2025), and HyperDet (Li et al., 2026a), the hypergraph acts as a feature-enhancement or cross-modal interaction component, and its output is consumed as an ordinary feature map. HC-MFM routes that output into the generation of the fusion weights instead. The C3 configuration isolates precisely this difference, and the accuracy it costs is the clearest evidence that the coupling, rather than the presence of a hypergraph module, is what carries the contribution of this level.

Within the weed-detection literature, the closest comparators are recent RT-DETR variants. PHRF-RTDETR (Jin et al., 2025) targets rice fields, EMCF-RTDETR (Yang et al., 2025) adds efficient multi-scale channel enhancement, and WS-RTDT (He et al., 2025) improves crop–weed discrimination through wavelet convolution and sparse cubic attention; WeedNet-R (Guo et al., 2023b) strengthens multi-scale features within a RetinaNet framework on sugar beet fields. These works improve the representation that is fed to the fusion stage; the present work changes what governs the fusion stage itself. The two directions are not in competition, and the relatively modest parameter cost of HC-MFM suggests that the conditioning mechanism could be transplanted onto such backbones. A second difference concerns evaluation: most of these studies report source-domain accuracy only. The cross-dataset results in Section 3.5 show that a detector can lead on the source benchmark and still lose roughly 44% of its accuracy on an unseen sugar beet field, which suggests that source-domain leaderboards alone are an incomplete basis for comparing field-deployable weed detectors.

The comparison with the higher-capacity baselines is consistent with this reading. Faster R-CNN and RetinaNet carry more parameters and computation than YOLOv11m yet reach a lower mAP50-95, indicating that in fine-grained detection of dense, highly similar objects, stacking convolutions and enlarging the receptive field does not by itself improve discriminability. The same pattern recurs across domains: D-FINE-L, YOLO11-X, and RF-DETR-L carry approximately 1.4, 2.7, and 5.9 times the parameters of ASSET-DETR and do not retain a larger share of their source-domain accuracy on the external sets. These observations are point estimates under a single protocol and should not be read as a general ranking, but they are consistent with the premise of this work that relational judgment, rather than capacity, is the binding constraint in this task.

### 4.3 Failure modes and remedies

Because the three categories arise from different causes, the remedies are correspondingly different. The scarcity of heavily overlapping and heavily occluded instances in the training set is the primary cause of the two occlusion categories, and instance-level augmentation such as Copy-Paste and CutMix-Object offers a direct means of enlarging this part of the sample distribution. The fixed connectivity of the hyperedges is a second factor under occlusion, since a node that carries only a fragment of the target is grouped by the same distance criterion as a fully visible one; letting the hyperedge connections vary adaptively with the features, that is, deformable hyperedge construction, would allow the grouping to follow the visible evidence. For the very-small-object category the limitation is one of resolution rather than of supervision, and slicing-based inference or a higher input resolution, optionally with a dedicated finer-scale detection branch, addresses it more directly than additional annotation. Weakly supervised consistency regularization is complementary to all three, in that it reduces the annotation cost of enlarging the occluded-instance pool. Reducing this annotation burden is itself an active direction: weakly supervised schemes that learn from scribble-level labels through cross-branch consistency (Han et al., 2026) suggest a route to enlarging the occluded-instance training pool without dense box annotation.

### 4.4 Cross-domain degradation and few-shot adaptation

Two features of the few-shot results require care in interpretation. First, under the 5% and 10% settings the target-domain mAP50 (0.815, 0.852) already exceeds the 0.742 of the source-domain test set, i.e., the retention rate exceeds 100%; this does not mean that the two test sets are of equal difficulty, nor can it alone prove that the domain shift has been fully eliminated, but mainly reflects that the relatively limited class distribution of OD-SugarBeets is more easily fit under a small amount of supervision. Therefore, the few-shot results should be understood as evidence of recovery under the present target-domain protocol, rather than a generalization guarantee for all sugar beet field scenes.

The class-level results in **Supplementary Table 3** further indicate that cross-domain degradation is not an overall accuracy scaling, but may be related to differences in target-domain class appearance, scale, and imaging conditions (dominated by the aforementioned weed-class object-discovery/localization failure); the present diagnosis does not show that a difference in annotation definition is the sole dominant factor. The drop on OD-SugarBeets is concentrated in the weed class, whereas both classes lose on PhenoBench. Therefore, under the present data and evaluation protocol, the results suggest that ASSET-DETR has a certain relative transfer and few-shot adaptation potential on external domains; these results should be understood as evidence under the present protocol rather than a generalization guarantee for all sugar beet field scenes, and they are not yet sufficient to determine the independent contribution of specific modules to cross-domain performance—stronger mechanistic conclusions still require target-domain module ablation and same-image qualitative comparison.

In addition, although the 1% and 5% few-shot settings report the mean and standard deviation over 3 support sets, each set is optimized only once under a fixed setting with random seed 0, and the randomness of support sampling and parameter optimization has not yet been separated; the 10% setting remains a point estimate of a single support set, and its stability awaits verification with more independent sampling.

### 4.5 Limitations and deployment considerations

One limitation concerns deployment. The efficiency figures reported in Section 3.1 were obtained on a workstation-class GPU, whereas the precision weeding and variable-rate spraying scenarios that motivate this work are typically served by embedded modules whose compute throughput and memory bandwidth are one to two orders of magnitude lower. Such hardware was not available during this study, and rather than extrapolate device figures we identify where the architecture is most likely to be constrained.

The hypergraph computation stage is the principal candidate. Because every node induces one hyperedge, the incidence matrix is square in the node count, and both its construction and the two propagation stages carry a cost that grows quadratically with the node count and therefore with the input area, while the matrix itself occupies roughly 82 MB in single precision at a batch size of eight. On a bandwidth-limited device this stage, rather than the convolutional backbone, is expected to dominate; restricting hypergraph-conditioned fusion to the coarser pyramid levels, reducing the aggregation resolution, or sparsifying the incidence matrix are the natural mitigations. The multi-scale attention in CAG-Backbone is a secondary candidate, since its key and value paths are evaluated at several dilation rates before pooling. On-device latency, thermal behaviour, power draw, and post-quantization accuracy therefore remain to be measured.

The 21.34 M parameters of ASSET-DETR correspond to approximately 85.4 MB of FP32 weights, which can be compressed to about 42.7/21.3 MB by FP16/INT8 quantization, and its core operators are supported by ONNX, TensorRT, OpenVINO, and the like, so the export path itself is not the obstacle.

## 5 Conclusion

Detecting weeds among sugar beet seedlings requires accuracy and efficiency at once, under morphological similarity between crop and weed, wide scale variation, and heterogeneous backgrounds. We addressed this combination by rebuilding RT-DETR at three levels into ASSET-DETR, whose central methodological contribution is HC-MFM: the group-level semantics recovered by hypergraph computation condition the generation of the multi-scale fusion weights, turning fusion from aggregation by local statistics into relation-conditioned feature arbitration.

On the public Lincoln Beet benchmark ASSET-DETR attains 74.2% mAP50 and 51.7% mAP50-95, exceeding twelve representative detectors under a unified protocol while running in real time at 100 FPS. The model is therefore a compact candidate for object-level perception in variable-rate spraying and precision weeding. Two limitations bound these conclusions: zero-shot accuracy on unseen sugar beet fields falls to about 56% of the source-domain level, although a small amount of target-domain annotation largely restores it, and the efficiency figures were obtained on a workstation GPU rather than on the embedded hardware that field deployment would use. On-device validation and adaptive, deformable hyperedge construction are the natural next steps.

## Data Availability

Publicly available datasets were analyzed in this study. These data can be found at the following locations: Lincoln Beet (LB): https://github.com/LAR/lincolnbeet_dataset; direct download: https://www.dropbox.com/s/0rq7cc8t6rja632/all_fields_lincolnbeet.zip?dl=0; OD-SugarBeets/SugarBeets2016: https://www.ipb.uni-bonn.de/data/sugarbeets2016/index.html; dataset directory: https://www.ipb.uni-bonn.de/datasets_IJRR2017/; PhenoBench: https://www.phenobench.org/dataset.html; direct download: https://www.phenobench.org/data/PhenoBench-v110.zip.
