# Supplementary material for: ASSET-DETR: a relation-aware multi-scale feature arbitration network for real-time weed detection in sugar beet fields
Source: Front Plant Sci. 2026 Aug 31;17:1931798. doi: 10.3389/fpls.2026.1931798 (PMC13572428; doi:10.3389/fpls.2026.1931798)
Supplement: Supplementary file 1 [file DataSheet1.docx]

Supplementary Material

**Contents**

**S1.** Full few-shot target-domain adaptation results on OD-SugarBeets (Table S1) — supports Section 3.5.3.

**S2.** Cross-model comparison under 5% few-shot adaptation on OD-SugarBeets (Table S2) — supports Section 3.5.4.

**S3.** Class-level cross-dataset generalization analysis (Table S3) — supports Section 3.5.2 and the Discussion.

**Supplementary Figures.** Figures S1–S6: dataset samples, distribution and ranking comparisons, marginal efficiency, ablation-progress curve, and failure cases.

**Supplementary Tables.** Tables S4–S8: experimental configuration, external-dataset statistics, cross-model cross-dataset details, and internal ablation details.

# S1 Full Few-Shot Target-Domain Adaptation Results

This section provides the complete per-protocol results of the few-shot target-domain adaptation experiment of ASSET-DETR on the external OD-SugarBeets dataset (Table S1), complementing the summary reported in Section 3.5.3 of the main text. The 1% and 5% settings are built on three independent support sets constructed with sampling seeds 42, 123, and 2024; results are reported as mean ± sample standard deviation, and the model training random seed is fixed to 0 for every support run. The 10% setting currently uses a single support set (sampling seed 42) and is therefore reported as a single point estimate.

**Table S1.** Few-shot target-domain adaptation results on OD-SugarBeets.

| **Protocol** | **Train images** | **Train boxes** | **P** | **R** | **F1** | **mAP50** | **mAP50-95** | **Sugar beet mAP50** | **Weed mAP50** |
| --- | --- | --- | --- | --- | --- | --- | --- | --- | --- |
| Zero-shot | 0 | 0 | 0.499 | 0.383 | 0.433 | 0.374 | 0.210 | 0.709 | 0.039 |
| Few-shot 1% | 35 | 115 | 0.771 ± 0.037 | 0.589 ± 0.094 | 0.668 | 0.657 ± 0.046 | 0.375 ± 0.019 | 0.892 ± 0.003 | 0.421 ± 0.092 |
| Few-shot 5% | 173 | 606 | 0.826 ± 0.005 | 0.762 ± 0.015 | 0.793 | 0.815 ± 0.009 | 0.480 ± 0.004 | 0.941 ± 0.003 | 0.690 ± 0.021 |
| Few-shot 10% | 347 | 1,269 | 0.849 | 0.799 | 0.823 | 0.852 | 0.501 | 0.944 | 0.761 |

*Note:* the 1% and 5% results are reported as mean ± sample standard deviation over three independent support sets (seeds 42/123/2024); the 10% result is a point estimate from a single support set; the zero-shot setting uses no target-domain training sample.

# S2 Cross-Model Comparison under 5% Few-Shot Adaptation

This section reports the full comparison of ASSET-DETR against four strong baseline detectors in terms of few-shot adaptation ability under 5% target-domain annotation on OD-SugarBeets (Table S2), complementing Section 3.5.4 of the main text. All baselines are trained and evaluated under exactly the same protocol as ASSET-DETR. Under the present 5% support setting, ASSET-DETR attains the largest gain (+0.441) and the highest retention rate (109.8%), ahead of RF-DETR-L (107.3%), D-FINE-L (105.9%), and YOLO11-X (104.2%), with RT-DETR-R18 the lowest (98.5%).

**Table S2.** Cross-model comparison of 5% few-shot target-domain adaptation on OD-SugarBeets.

| **Model** | **Source** | **Zero-shot OD** | **5% few-shot OD** | **Gain** | **Retention rate** |
| --- | --- | --- | --- | --- | --- |
| RT-DETR-R18 | 0.664 | 0.322 | 0.654 | 0.332 | 98.5% |
| YOLO11-X | 0.733 | 0.344 | 0.764 | 0.420 | 104.2% |
| D-FINE-L | 0.746 | 0.365 | 0.790 | 0.425 | 105.9% |
| RF-DETR-L | 0.749 | 0.372 | 0.804 | 0.432 | 107.3% |
| ASSET-DETR | 0.742 | 0.374 | 0.815 | 0.441 | 109.8% |

*Note:* gain = (5% few-shot) − (zero-shot); retention rate = (5% few-shot) / (Source). The ASSET-DETR values are the mean over three support sets (seeds 42/123/2024).

# S3 Class-Level Cross-Dataset Generalization Analysis

This section provides the complete class-level statistics of cross-dataset generalization (Table S3), supporting the discussion in Section 3.5.2 and the Discussion of the main text. The results indicate that cross-domain degradation is not a uniform overall-accuracy scaling but arises mainly from differences in target-domain class appearance, scale, and imaging conditions: on OD-SugarBeets the drop is concentrated in the weed class, whereas on PhenoBench both classes lose to different degrees.

**Table S3.** Class-level cross-dataset generalization analysis.

| **Dataset / protocol** | **Sugar beet mAP50** | **Sugar beet mAP50-95** | **Weed mAP50** | **Weed mAP50-95** | **Main observation** |
| --- | --- | --- | --- | --- | --- |
| Source test | 0.809 | 0.649 | 0.675 | 0.385 | Both classes remain well detected within the source domain. |
| OD zero-shot | 0.709 | 0.411 | 0.039 | 0.010 | Cross-domain drop is driven by the collapse of the weed class. |
| OD 10% few-shot | 0.944 | 0.661 | 0.761 | 0.342 | Few-shot fine-tuning mainly restores weed-class detection. |
| PhenoBench zero-shot | 0.598 | 0.499 | 0.306 | 0.158 | Both classes drop, but the weed class does not nearly collapse. |

*Note:* this table is used to explain the class-dependent generalization differences observed across the different datasets.

# Supplementary Figures

**Figure S1.** Representative samples of the Lincoln Beet (LB) dataset.

**Figure S2.** Distribution comparison of ASSET-DETR and the baseline detectors over six metrics (the red band indicates the lead over the relatively best baseline).

**Figure S3.** Comparison of the ranking trajectories of the detectors over the six detection metrics.

**Figure S4.** Comparison of the computation increment and accuracy gain of each ablation configuration relative to the baseline (Exp. 1) (the rightmost column is the marginal efficiency, in pp/GFLOP).

**Figure S5.** Typical failure cases of the zero-shot detection of the weed class by ASSET-DETR on OD-SugarBeets (left: ground truth; right: zero-shot prediction, confidence threshold 0.25).

**Figure S6.** Progressive changes in detection performance across the combination configurations in the ablation study.

# Supplementary Tables

**Table S4.** Experimental hardware/software environment and training hyperparameter configuration.

| **Item** | **Configuration / Version** |
| --- | --- |
| GPU | NVIDIA vGPU-32GB (partitioned from an A100, 32 GB memory) × 1 |
| CPU | Intel(R) Xeon(R) Platinum 8352V @ 2.10 GHz (16 vCPU) |
| Memory | 62 GB |
| Disk | 30 GB system disk + 50 GB data-disk SSD |
| Operating system | Ubuntu 20.04 LTS |
| Deep-learning framework | PyTorch 2.0.0 |
| CUDA version | CUDA 11.8 |
| Programming language | Python 3.8 |
| Optimizer | AdamW (lr = 1×10⁻⁴, weight decay = 1×10⁻⁴) |
| Learning-rate schedule | Linear warm-up (5 epochs) + Cosine annealing |
| Input resolution | 640 × 640 |
| Batch size | 8 (gradient accumulation step = 2, effective batch size = 16) |
| Training epochs | 300 (early-stop patience = 30) |
| Random seed | 42 for the source-domain data split; training seed 0 for point-estimate runs; training seeds 0/1/2 (mean ± SD) for the key internal ablations; few-shot: support-set sampling seeds 42/123/2024 with training seed 0 for the 1% and 5% settings, and sampling seed 42 with training seed 0 for the 10% setting. |

**Table S5.** Statistics and annotation-conversion rules of the external generalization datasets.

| **Dataset** | **Annotation format** | **Image split** | **Evaluation set** | **Harmonized classes** | **No. of boxes** |
| --- | --- | --- | --- | --- | --- |
| OD-SugarBeets | VOC XML bbox | train 3,466 / val 385 / test 964 | Official test, 964 images | crop → sugar beet; weed → weed | train 13,391 / val 1,509 / test 3,868 |
| PhenoBench | semantics + plant_instances | train 1,407 / val 772 | Official val as test, 772 images | crop/partial-crop → sugar beet; weed/partial-weed → weed | train 19,862 / val-test 10,341 |

**Table S6.** Cross-model zero-shot cross-dataset generalization comparison.

| **Model** | **Source** | **OD-SugarBeets** | **PhenoBench** | **Mean target-domain** | **ΔmAP** | **Retention rate** |
| --- | --- | --- | --- | --- | --- | --- |
| RT-DETR-R18 | 0.664 | 0.322 | 0.386 | 0.354 | −0.310 | 53.3% |
| YOLO11-X | 0.733 | 0.344 | 0.419 | 0.381 | −0.352 | 52.0% |
| D-FINE-L | 0.746 | 0.365 | 0.442 | 0.404 | −0.342 | 54.2% |
| RF-DETR-L | 0.749 | 0.372 | 0.447 | 0.409 | −0.340 | 54.6% |
| ASSET-DETR | 0.742 | 0.374 | 0.452 | 0.413 | −0.329 | 55.7% |

**Table S7.** Internal ablation of the CAG-Backbone.

| **Configuration** | **mAP50 (%)** | **mAP50-95 (%)** | **Params** |
| --- | --- | --- | --- |
| B0 Full | 74.2 ± 0.3 | 51.7 ± 0.4 | 21.34 |
| B1 w/o multi-scale | 71.9 ± 0.4* | 49.3 ± 0.5* | 19.9 |
| B2 Dilation {2,4,6} | 73.7 ± 0.3* | 51.2 ± 0.3* | 20.2 |
| B3 w/o CGLU | 72.9 ± 0.3* | 50.4 ± 0.4* | 19.7 |

*Paired t-test versus the full model, p < 0.05; Params are in millions (M).

**Table S8.** Internal ablation of HyperFPN (C3 is the key comparison).

| **Configuration** | **mAP50 (%)** | **mAP50-95 (%)** | **Params** |
| --- | --- | --- | --- |
| C0 Full | 74.2 ± 0.3 | 51.7 ± 0.4 | 21.34 |
| C1 Smaller τ than C0 | 73.6 ± 0.2* | 51.0 ± 0.3* | 21.34 |
| C2 Larger τ than C0 | 73.8 ± 0.2 | 51.2 ± 0.3* | 21.34 |
| C3 Standard MFM | 73.5 ± 0.3* | 50.9 ± 0.3* | 20.15 |

*Paired t-test versus the full model, p < 0.05; Params are in millions (M).
